# Proton Transfer through a Charged Conduit in Respiratory Complex I: Long-Range Effects and Conformational Gating

**DOI:** 10.1021/acs.jcim.5c01365

**Published:** 2025-09-18

**Authors:** Luka Simsive, Oleksii Zdorevskyi, Vivek Sharma

**Affiliations:** 1 Department of Physics, 3835 University of Helsinki, Helsinki 00014, Finland; 2 HiLIFE Institute of Biotechnology, 3835 University of Helsinki, Helsinki 00014, Finland

## Abstract

Energy coupling processes in respiratory complex I, a large redox-driven proton pump in the inner mitochondrial membrane, remain one of the most enigmatic problems in modern bioenergetics. Recent high-resolution cryo-EM structures of complex I revealed extensive hydration in the interior of the protein, including the buried E channel, which is an acidic charged conduit that bridges the quinone binding cavity with the extended membrane domain of the enzyme. Despite the general agreement that E channel participates in proton transfer, the absence of proton density in the cryo-EM maps poses a significant challenge to develop viable models of proton pumping. By adhering to the hypothesis that E channel catalyzes transfer of proton(s) from the quinone binding cavity to the membrane-bound proton pumping site(s), we performed hybrid quantum mechanics/molecular mechanics (QM/MM) molecular dynamics (MD) simulations using the ∼2.4 Å cryo-EM structure of mitochondrial complex I from*Mus musculus*. By combining classical atomistic MD simulations with hybrid QM/MM free energy calculations, we identify several energetically favorable Grotthuss-competent proton transfer paths in the E channel region. As part of the long-range coupling in complex I, our calculations show that protonation of a single acidic amino acid residue in the distal MM surroundings can alter the dynamics of proton transfer in the E channel region. Additionally, we pinpoint the gating function of a highly conserved tyrosine residue in the E channel, which undergoes conformational flipping to establish an energetically favorable proton transfer path. In the context of the redox-coupled proton pumping mechanism of complex I, we propose a stepping-stone model of proton transfer through the E channel.

## Graphical abstract

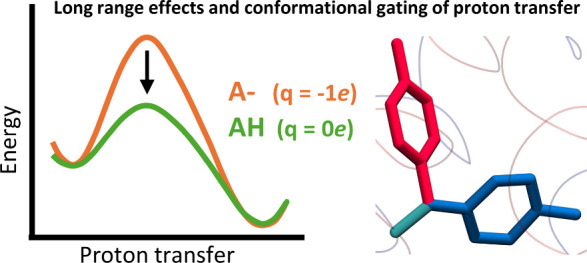

## Introduction

Proton (H^+^) transfer is a fundamental reaction in chemistry and biology. With a diffusion coefficient of 10^–5^ cm^2^/s in bulk water,^1^ an excess proton can diffuse by ∼10 Å in a short time span of ∼100 picoseconds (ps). The fast rate of proton diffusion is due to the Grotthuss mechanism of proton transfer in which a proton jumps (hops) between hydrogen-bonded water molecules in ps time scales and can travel long distances.^1^ State-of-the-art ab initio and reactive molecular dynamics (MD) simulations as well as spectroscopic measurements have identified Zundel and Eigen species as metastable cationic intermediates^2,3^ that form during the transfer of an excess proton in bulk water. Another propagating defect is the transport of the hydroxide (−OH^–^) ion. However, properties of its dynamics and energetics are more challenging to understand in comparison to excess proton transfer.^4^ In the absence of an excess proton, the propagation of H^+^ through a hydrogen-bonded water wire is equivalent to the propagation of OH^–^ in the opposite direction.

Various photosynthetic and respiratory enzymes also catalyze long-range proton transfer reactions as part of their core energy-generating function.^5–7^ During enzymatic proton transfer, protons hop between polar amino acid residues and water molecules via Grotthuss-like mechanism often involving coupling to some redox and/or conformational changes.^8–10^ It can be challenging to identify if the enzymatic proton transfer occurs as the transport of an excess proton or charge separation.^11–14^ The high-resolution 3D structures of bioenergetic protein complexes have revealed paths consisting of water molecules and polar amino acids, which are often taken as a proxy for proton transfer. But the question of whether proton transfer actually occurs through the region remains challenging to decipher. Protons (unlike electrons) cannot tunnel for long distances. Already, a gap of about 5–6 Å between the two protonatable groups would make proton transfer unfeasible between them, unless they are bridged by a water molecule(s) and/or other protonatable groups such as amino acid side chains.

The large respiratory enzyme complex I functions as a redox-driven proton pump in mitochondria and many bacteria^15–18^ (Figure 1). By pumping protons, it contributes to proton electrochemical gradient across the membrane, which is utilized to drive ATP production and metabolite transport.^18^ The pumping of protons by complex I is coupled to the reduction of quinone (Q) that binds in an ∼30 Å long chamber (Figure 1). However, the molecular mechanism of ubiquinone reduction and proton translocation remains unclear.^19–23^ In several high-resolution cryo-EM structures of complex I, water-filled regions surrounded by polar amino acid residues were identified,^15–17^ which led to the conclusion that protons can be transported through these hydrated pathways. One such highly conserved region is the E channel region (Figure 1), a ∼35 Å long and 4–6 Å wide^24^ conduit formed by acidic amino acid residues, in particular glutamic acids. This channel, which connects the quinone tunnel with the membrane arm of the complex, is found hydrated in several high-resolution cryo-EM structures of complex I and has been suggested to be a key element in coupling the redox reactions to proton pumping.^18^

**Figure 1:**
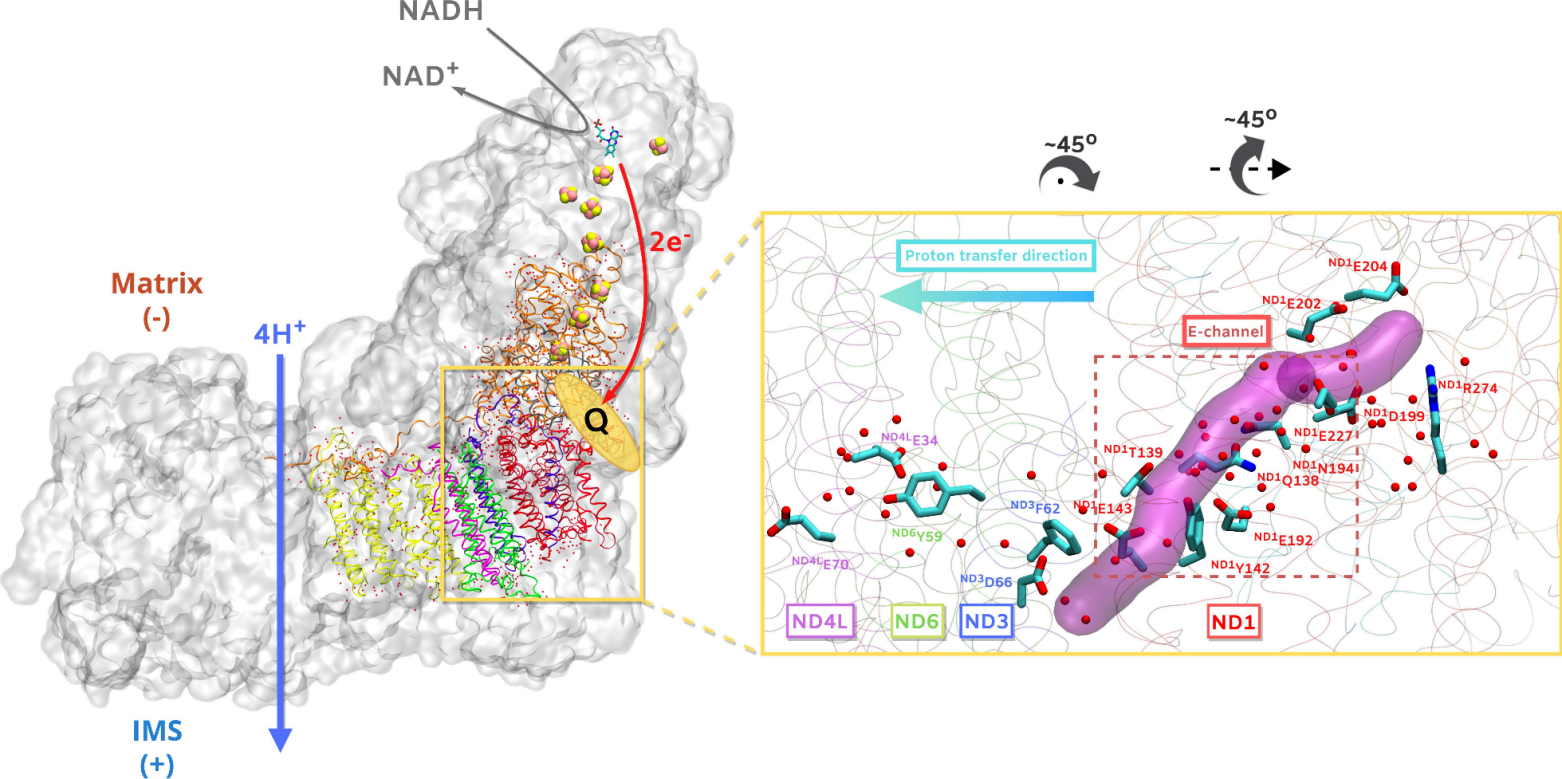
Mitochondrial complex I from*Mus musculus*­(PDB 8OM1) shown in transparent gray surface (left). NADH oxidation at the end of the peripheral arm of complex I releases electrons (red curved arrow) that travel via FMN (sticks) and FeS clusters (yellow/pink spheres) to quinone (Q) bound in a ∼30 Å long Q cavity (yellow colored ellipse). Two-electron reduction of Q drives proton pumping (blue arrow) in the extended membrane arm of complex I. Selected core subunits surrounding the E channel region and included in our model systems are shown with ribbons. Structurally resolved water molecules in these subunits are shown as red spheres. (Right) The E channel (dotted frame), and the conduit passing through it is highlighted in magenta transparent surface. Structurally resolved water molecules are shown as red spheres. Key amino acid residues are displayed with sticks and labeled according to the subunit they belong to. IMS – inter membrane space. Subunits shown in the left panel are ND2 (yellow), ND4L (purple), ND3 (blue), ND1 (red), ND6 (green), NDUFS2 (orange), and NDUFS7 (gray).

Early on based on a ∼3.3 Å X-ray structure of*Thermus thermophilus*complex I, proton pumping function of the E channel was proposed.^25^ Large-scale atomistic classical MD simulations of entire bacterial complex I structure from *T. thermophilus* showed extensive hydration of the E channel region.^26,27^ The simulation data demonstrated that water molecules in the E channel region align to yield a net dipole moment with the direction of the vector pointing from the Q tunnel region to the membrane domain of the complex.^27^ Classical MD simulations of a higher-resolution (∼2.4 Å) *Yarrowia lipolytica* complex I structure in a charge state determined based on p*K*_a_ calculations showed that the hydration of the E channel depends on the protonation (and conformational) states of acidic residues.^17^ Building upon recent high-resolution cryo-EM structural data in different catalytic states, Sazanov and colleagues have postulated a mechanism of proton pumping in which proton transfer occurs from the membrane domain towards the Q tunnel via E channel.^21^ The mechanism shares similarity with the “electrostatic domino” effect proposed by Hummer and Wikström in which negative charges created in the Q tunnel are propagated to the membrane domain via E channel.^23^ Given the central importance of the E channel in driving redox-coupled proton pumping, Djurabekova et al. have highlighted the importance of charge state of E channel in the active/deactive (A/D) transition of complex I.^19^ Kaila and colleagues analyzed hydration and protonation energetics of the E channel region in the A/D states of complex I with multiscale simulation approaches, but somewhat higher activation energy barriers were obtained.^10,28^ By performing QM/MM simulations, Haapanen et al. studied the transfer of protons upon quinol oxidation to the acidic residues in the vicinity of the E channel,^29^ but no quantitative energetics were obtained. More recently, by applying continuum electrostatics combined with classical MD simulations, a direct role of E channel in proton pumping has been suggested.^30^ Additionally, using Dowser-based approaches on cryo-EM structures,^31–33^ a higher-level of hydration in the E channel of complex I was predicted,^24^ a notion that is also commensurate with the earlier findings based on structures and classical MD simulations.^17,34^

Despite a wealth of high-resolution structural data on complex I, the protonation state of the E channel residues as well as their surroundings remain unclear (cf.^35,36^ ). What is also unknown is the direction of the proton transfer during complex I turnover; does it occur from the Q tunnel toward the extended membrane domain or other way around(?). Additionally, the origin and fate of protons traveling through the E channel is not known, that is, if they are substrate or pumped protons. These are all extremely challenging questions to answer from both computational and experimental points of view. In this work, we probe the energetics of proton transfer through the E channel region in a quantitative manner using hybrid QM/MM free energy simulations on the high-resolution structure (∼2.4 Å) of mitochondrial complex I from*Mus musculus* (PDB 8OM1).^16^ This cryo-EM structure is trapped in an active resting state of the enzyme and displays one of the highest levels of hydration in the E channel (Figure 1B). By adhering to the hypothesis that proton transfer occurs from the Q tunnel toward the membrane domain of complex I,^19^ the results from our multiscale simulations show that protons can diffuse in the E channel with low energetic costs. We also demonstrate that long-range effects play an important role in driving proton transfer along the pathway formed by water molecules and polar amino acid residues. We discuss energetically feasible pathways of proton transfer and show proton gating by a conserved tyrosine residue that undergoes conformational changes. Overall, our results align with the recent mechanistic investigations on complex I^19^ and support the role of the E channel in catalyzing proton transfer as part of the proton pumping mechanism of complex I.

## Results

### Ground Level Charge State of the Protein

The results of conventional classical and QM/MM MD simulations depend on several factors,^37,38^ one of them being the selected protonation states of titratable residues in the MM region. Due to the presence of several titratable sites in the MM region of our QM/MM model (around 10), we decided to initiate our QM/MM calculations with the charge state of the protein determined from empirical p*K*_a_ calculations (see Methods). It is known that such p*K*_a_ estimates are approximate in nature; however, they can yield reasonable proton affinity estimates especially for buried amino acid residues,^39^ which is the case here (see also refs 40 and 41 and Table S1). Furthermore, when classical atomistic MD simulations of high-resolution cryo-EM structures are performed in charge states determined by empirical p*K*_a_ calculations, relatively stable backbone and side chain conformations have been obtained.^35^ It has also been shown previously that empirical p*K*_a_ estimates of several acidic residues of complex I agree with the charge state predictions based on the cryo-EM density analysis.^36^ Building upon these arguments, we decided to launch our unbiased and biased QM/MM MD simulations with the charges of MM residues determined by empirical p*K*_a_ estimates. We then systematically altered the protonation states of several residues in the MM and QM regions in a step-by-step manner (see Methods, Tables S2–S6).

To model the long-range proton transfer from the E channel/Q tunnel junction toward the extended membrane domain of complex I (Figure 1), we split the entire E channel and its surroundings into smaller subsections (Figure S1, Tables S2–S6). We then constructed several different QM regions based on these subsections that span the shallow region of the quinone binding pocket (near transmembrane helix (TMH) 5-6 loop region), core of the E channel, and all the way to the ND6/ND4L interface, marking the beginning of the horizontal hydrophilic axis embedded in antiporter-like subunits (see Figure S1).

### Long-Range Effects: Charge Change in Distal MM Region Alters Proton Transfer Energetics

We first studied the proton transfer reactions in the ^ND3^Asp66–^ND4L^Glu34 section (Figure S1B), which resides at the end of the E channel region toward the extended membrane domain of complex I. This region has been found to be functionally central and undergoes conformational transitions in the open/closed (deactive/active) catalytic states of the enzyme.^15–17,21,28,42^ In the cryo-EM structure used for QM/MM modeling, TMH3 of ND6 is modeled in the α-helical conformation. This has been shown to open a water-based route between the E channel region and the extended hydrophilic axis of the membrane domain of complex I, which is otherwise blocked in the π helical conformation of TMH3 (see also ref 17). Due to the absence of the Grotthuss-competent water network in the cryo-EM structure, we carried out an additional classical MD simulation in the charge state determined by p*K*_a_ estimates (see Methods). This provided essential hydration in the region leading to the formation of a three-water bridge between ^ND3^Asp66 and ^ND4L^Glu34 (Figure S2).

In the classically equilibrated ground state (setup L0; Table S2), we did not observe any spontaneous protonation/deprotonation reactions. Therefore, we created a proton vacancy on ^ND4L^Glu34 (in the QM region) to drive the Grotthuss “hole”-type proton transfer (setup L1, Table S2) and investigated possible proton relay from the protonated ^ND3^Asp66 to anionic ^ND4L^Glu34 through three-water molecules (Figure S2). The unbiased QM/MM MD simulation did not show any spontaneous proton transfer between ^ND3^Asp66 and ^ND4L^Glu34, highlighting the necessity to probe the free energy landscape with umbrella sampling simulations (see Methods). Our QM/MM free energy simulations with p*K*_a_ calculation-based protonation states yielded an activation energy barrier of ∼11 kcal/mol and an endergonic behavior (ca. 4 kcal/mol) of proton transfer between ^ND3^Asp66 and ^ND4L^Glu34 (Figure 2, left panel), leading us to create additional states in which charges were altered in the MM and the QM regions.

**Figure 2:**
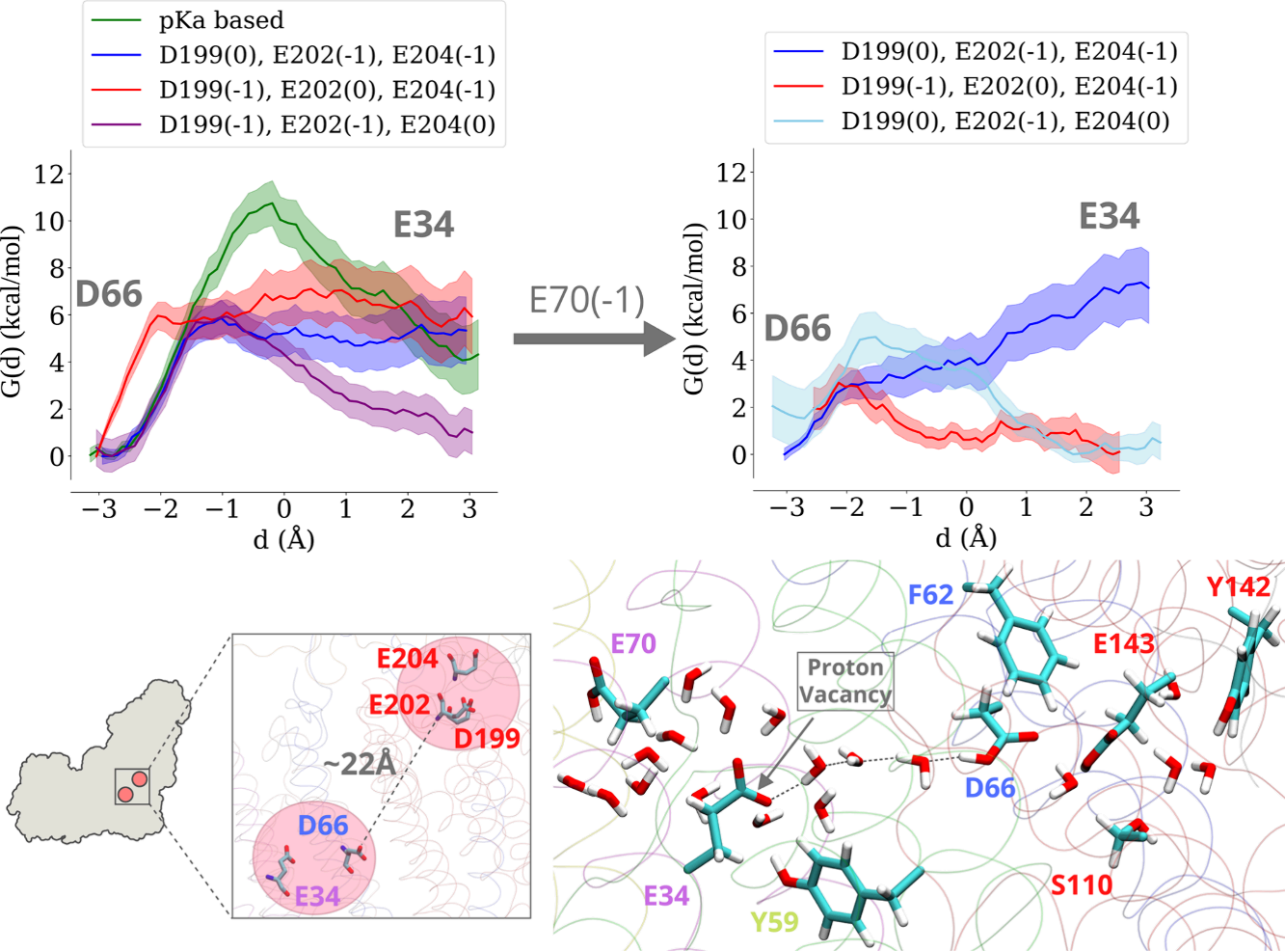
Long-range effects on proton transfer energetics between ^ND3^Asp66 and ^ND4L^Glu34. Neutralization of acidic residues ^ND1^Asp199, ^ND1^Glu202, or ^ND1^Glu204 of the TMH5-6 loop region changes the free energy, *G*(*d*), profile of proton transfer in the QM region in states when ^ND4L^Glu70 is modeled charge neutral (left panel) or anionic (E70(−1), right panel). The statistical error bars in free energy profiles are shown in different shades (see Methods). The location and separation of ^ND3^Asp66/^ND4L^Glu34 residues from ^ND1^Asp199/^ND1^Glu202/^ND1^Glu204 on TMH5-6 loop region is shown in the perspective of complex I (lower left panel), and the initial state of the QM region and proton vacancy position are shown in the lower right panel, with the gray dotted lines highlighting the proton transfer route studied.

The conserved acidic residue ^ND1^Asp199 in the MM region, located ca. ∼20 Å from the QM region, has been suggested to be a putative proton loading site in the catalytic mechanism of respiratory complex I^17,19^ (see also ref 43). Similar proton loading (storage/trap) sites are known to exist in other redox- and photoactive enzymes, e.g., cytochrome *c* oxidase^44,45^ and bacteriorhodopsin.^46,47^ To study the effect of proton loading on proton transfer energetics, we modeled acidic residue ^ND1^Asp199 as charge neutral (setup L2, Table S2) and observed that this dramatically reduces the activation energy barrier by 5–6 kcal/mol (Figure 2, left panel), highlighting a long-range electrostatic effect on the energetics of proton transfer. We then systematically neutralized the two other TMH5-6 loop region residues (Glu202 and Glu204 from the ND1 subunit; see Figure 1). The acidic residues on this dynamic loop are conserved in complex I family and are known to be functionally important.^48^ The data show that similar to the neutralization of ^ND1^Asp199, charge neutral states of ^ND1^Glu204 or ^ND1^Glu202 improve the free energy profile of proton transfer between ^ND3^Asp66 and ^ND4L^Glu34. Overall, the addition of one extra charge in the MM region (TMH 5-6 loop region) dramatically perturbs the protonation energetics in the QM region ca. 20 Å apart (Figure 2, top left panel). Since the number of water molecules can also perturb free energy profiles of proton transfer, we tested the effect of number of water molecules between ^ND3^Asp66 and ^ND4L^Glu34 (three vs four) on the proton transfer energetics and found that it is energetically favorable when proton transfer occurs via three instead of four water molecules (see Figure S3).

To further probe the energetics of proton transfer, an additional proton vacancy was created on ^ND4L^Glu70 (part of the QM region; see setup L3, Table S2). However, this resulted in a surprising disruption of the water wire between ^ND3^Asp66 and ^ND4L^Glu34 and observed in both QM/MM and classical MD simulations (Figure S4). A large gap of 5–6 Å in water wire connectivity renders the proton transfer between ^ND3^Asp66 and ^ND4L^Glu34 unfeasible. Therefore, we modeled the protonated state of the putative proton loading site ^ND1^Asp199 in the MM region, located more than 20 Å from the QM region, and observed that its neutralization stabilized the hydrogen-bonded water wire (Figure S4; setup L4, Table S2). Similar to the results described above, charge changes in the TMH5-6 loop region were found to alter the free energy profiles of proton transfer (Figure 2, left and right panels). The charge neutralization of ^ND1^Glu204 on the top of already protonated ^ND1^Asp199 (setup L6, Table S2) or of ^ND1^Glu202 alone (setup L5, Table S2) changed the energetics of proton transfer between ^ND3^Asp66 and ^ND4L^Glu34 (see Figure 2, right panel, cyan and red curves, respectively). These data highlight that long-range interactions can not only modify the energetics of proton transfer (Figure 2) but also the conformational dynamics of proton transfer paths (Figure S4).

Next, we investigated the proton transfer in the region between ^ND1^Glu143 and ^ND3^Asp66 (Figure S1). Since the anionic nature of ^ND4L^Glu70 perturbed the water wire dynamics (see above), we kept it charge neutral (as also predicted by p*K*_a_ calculations) in all subsequent QM/MM setups. In the cryo-EM structure of complex I, an ∼4 Å gap exists between ^ND1^Glu143 and ^ND3^Asp66, which is abridged upon structural relaxations during QM/MM minimization and dynamics (simulation snapshots shown in Figure S5). As a result, either a single water molecule mediates the putative proton transfer path between the two residues or alternatively the two residues come in direct hydrogen bonding contact with each other (Figure S5, right panels). We observed in our unbiased QM/MM MD simulations that proton transfer occurs rapidly from ^ND1^Glu143 to ^ND3^Asp66 within 1–3 ps under different conditions (Table S2, setups L8–L11), which is commensurate with the favorable free energy profile showing significantly low activation energy barriers especially with an additional proton modeled on putative proton loading site ^ND1^Asp199 (Figure 3, left panel). The QM/MM data suggest that the deprotonation of ^ND3^Asp66 by ^ND4L^Glu34 (see above) will result in spontaneous reprotonation of the aspartate from the neighboring ^ND1^Glu143 as a cascade effect either through a direct hydrogen bond or via an intervening water molecule. It is also noteworthy that deprotonation and protonation of ^ND3^Asp66 by ^ND4L^Glu34 and ^ND1^Glu143 occur on its two different oxygen atoms (OD2 and OD1), respectively (see Figure S6), with minimal displacement and without requiring the flipping of its carboxylate group or side chain.

**Figure 3:**
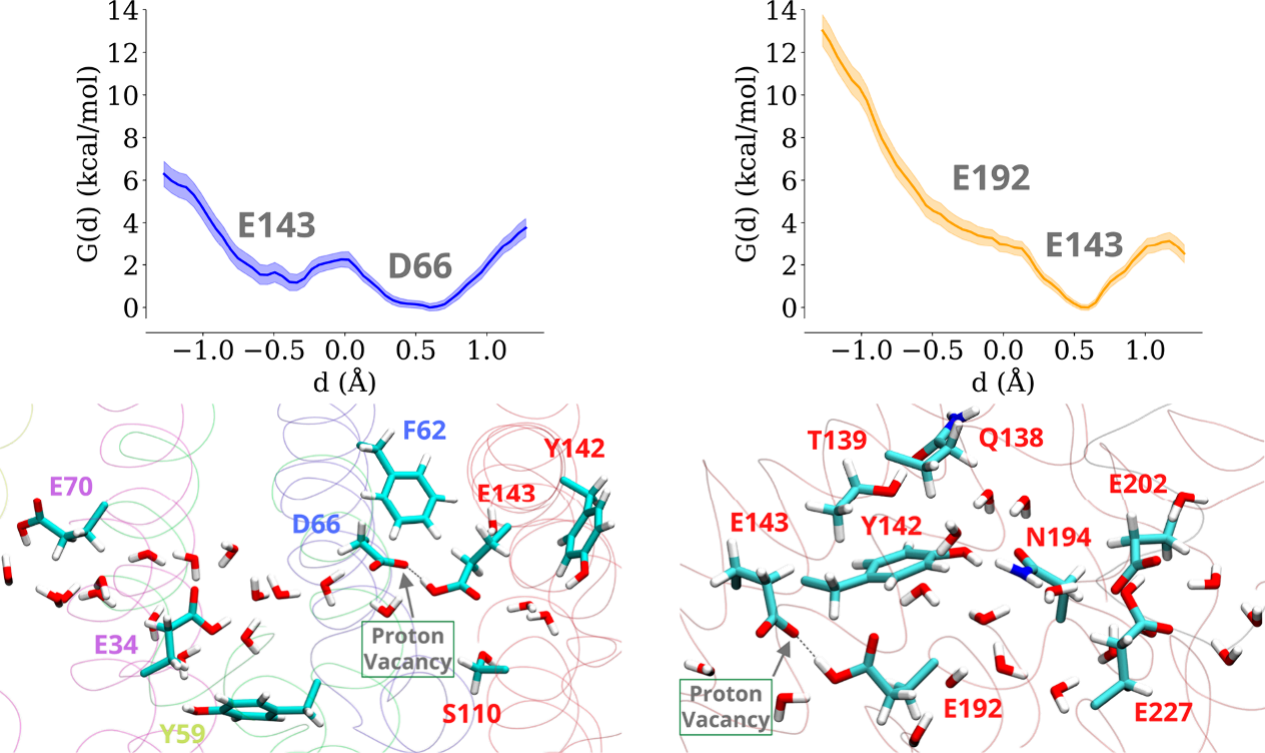
Energetically favorable proton transfer from ^ND1^Glu143 to ^ND3^Asp66 (left) and from ^ND1^Glu192 to ^ND1^Glu143 (right) with ^ND1^Asp199 in the MM region modeled as charge neutral (see also Figure 2). The ^ND1^Glu192–^ND1^Glu143 free energy profile is calculated from the configuration where ^ND1^Tyr142 is flipped (see the text). Shaded areas in the free energy profiles denote the bootstrapping errors sampled from the simulation windows (see Methods). The associated QM regions, the starting conformation, and the studied hydrogen-bonded paths are shown in the panels below.

### Proton Transfer through Conserved Tyrosine Is Unfavorable

Tyrosine residues in the membrane arm of complex I have been suggested to participate in proton transfer reactions. Previous QM/MM simulations on antiporter-like subunits showed that the transfer of proton through a tyrosine residue occurs in a highly concerted fashion; its deprotonation is immediately followed by reprotonation.^8^ However, our QM/MM simulations on the E channel of complex I show that the deprotonation of the highly conserved Tyr142 of the ND1 subunit is thermodynamically unfavorable (Figure 4). In several simulations when protonation states of surrounding residues in the MM region are perturbed, we find tyrosine deprotonation to be mostly endergonic (ca. 2–6 kcal/mol). For instance, when both ^ND3^Asp66 and ^ND3^Glu68 in the MM region (located ∼6 Å and ∼16 Å from ^ND1^Glu143, respectively, toward the extended membrane domain) are modeled in their anionic states (setup M4, Table S3), the deprotonated state of tyrosine is found to be energetically unfavorable with respect to its charge neutral state (Figure 4). Similarly, we neutralized the acidic residues of the TMH5-6 loop closer to the E channel/Q tunnel junction (Table S3, setups M4, M6, and M7), the energetics did not improve (Figure 4), which suggests that the proton affinity of tyrosine is significantly high and is not substantially perturbed upon charge changes in the MM region.

**Figure 4:**
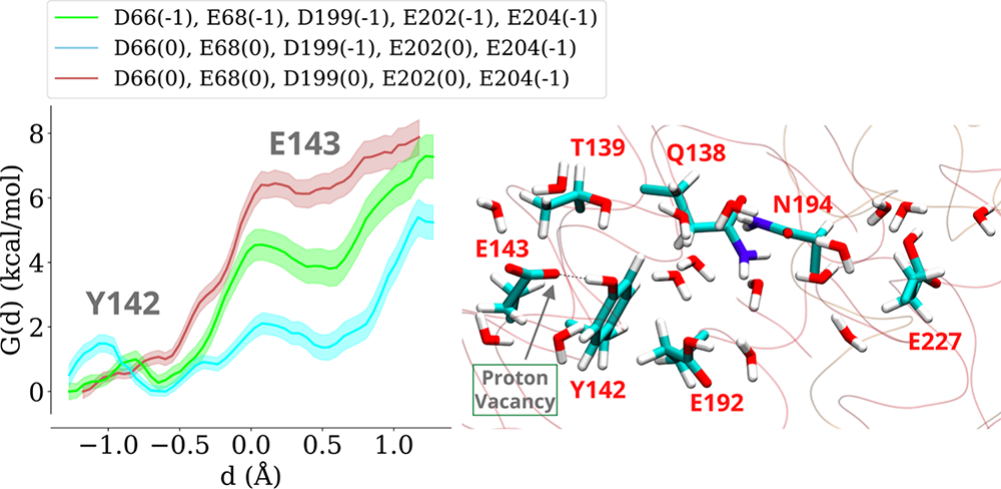
Proton transfer reactions through conserved tyrosine are energetically unfavorable. The three free energy profile (*G*(*d*)) plots (left) correspond to the different charge states of the MM region simulated (see text). The charge neutral and anionic states of ^ND1^Tyr142 correspond to *d* ca. −0.5 and +0.5 Å, respectively. The QM region with the gray dotted hydrogen bond shows the initial conformation and the studied proton transfer path (right). The bootstrapping errors are shown as shaded areas around the free energy profiles.

Given that proton transfer through tyrosine is energetically unfavorable, we also simulated the transfer of a proton from ^ND1^Glu192 to ^ND1^Glu143 bypassing the tyrosine residue but involving two water molecules (setups M10 and M11, Table S3). The results show that proton transfer is still unfavorable and the two end points (proton on ^ND1^Glu192 or ^ND1^Glu143) become isoenergetic only when all acidic amino acid residues of the TMH5-6 loop segment in the distal MM region are modeled charge neutral, which is an unlikely scenario (Figure S7, see also Tables S2 and S3, setup M11).

### Conserved Tyrosine as a Conformational Gate

Previous structural studies have shown that tyrosine residue can undergo a large conformational change upon activation and deactivation (and closed/open transitions) of the enzyme.^15,49,50^ Interestingly, our classical MD simulations on the active resting complex demonstrate two distinct orientations of ^ND1^Tyr142 (dihedral C-Cα-Cβ-Cγ ∼180 or ∼300°, Figure 5). In its flipped conformation (∼180°, closer to the open/deactive-like state, ∼153°), tyrosine hydrogen-bonds to residues ^ND1^Gln138/^ND1^Asn194. Though partly distinct from the configuration observed in the cryo-EM structure of the open state,^15^ the displacement of tyrosine creates a direct hydrogen bonding connection between ^ND1^Glu192 and ^ND1^Glu143, providing an alternative proton transfer route than via tyrosine or neighboring water molecules, as analyzed above. We next tested the direct proton transfer from ^ND1^Glu192 to ^ND1^Glu143 with the flipped tyrosine conformation and found that ^ND1^Glu192 rapidly (within 10 fs) donates the proton to its hydrogen-bonding partner ^ND1^Glu143. The proton bound to OE2 atom of ^ND1^Glu192 is transferred to the OE1 atom of ^ND1^Glu143, which is different from the oxygen atom of the latter residue that donates proton to ^ND1^Glu66 (see Figure S6). Umbrella sampling simulations (setups M1'–M4', Table S4) also show substantial stability of the protonated charge state of ^ND1^Glu143 (Figure 3, right panel). Overall, these findings point to the fact that ^ND1^Tyr142 may not be directly involved in proton transfer through the E channel, instead may act as a molecular gate, and enhance the proton traffic through this route by undergoing conformational change.^15,16^ So far, two main conclusions appear from the analysis above that the transfer of proton in the E channel can be enhanced by the conformational flip of the tyrosine side chain and by the protonation of distal TMH5-6 loop residues (e.g., ^ND1^Asp199, a putative proton loading site).

**Figure 5:**
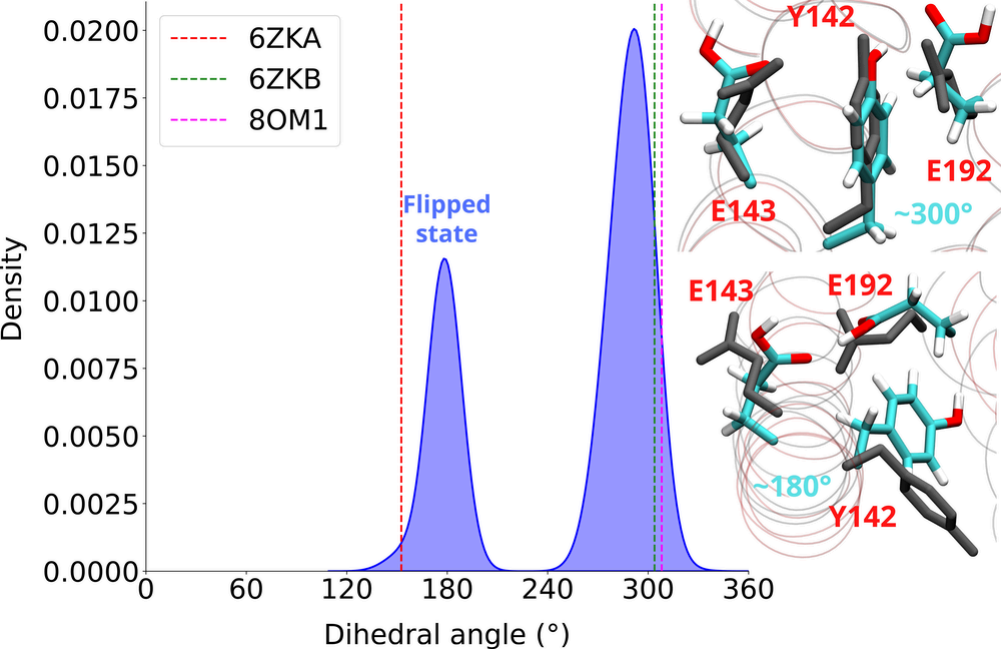
Conformational dynamics of tyrosine ^ND1^Tyr142. Classical MD simulations show the side chain of tyrosine undergoes conformational changes and occupies two states with dihedral C-Cα-Cβ-Cγ ∼180 or ∼ 300° (shown as distribution plot, left). The dotted lines show the structurally observed conformations (measured with the same dihedral) with PDB identifiers of the cryo-EM structures indicated. Structural changes in two conformations of tyrosine from MD simulations and cryo-EM data (right), with the cryo-EM conformations of amino acid side chains shown with gray sticks (see also Figure S8). The negative values of the dihedral angle (from −180 to 0°) are shifted by 360° to obtain a continuous distribution.

### Proton Transfer Reactions in the Q Tunnel/E Channel Vicinity

With the proton hole now at ^ND1^Glu192, we next studied the transfer of protons in the final section of the E channel that is from the conserved acidic residues of the TMH5-6 loop region (^ND1^Asp199, ^ND1^Glu202, ^ND1^Glu204 and ^ND1^Glu206) to the proton vacancy at ^ND1^Glu192. Since the transfer of proton from ^ND1^Glu192 to ^ND1^Glu143 with flipped tyrosine is spontaneous, all QM/MM models in this section were constructed with the tyrosine side chain flipped (see Tables S4 and S5). Furthermore, we note that TMH5-6 loop residue ^ND1^Glu202 is predicted to be anionic both based on p*K*_a_ calculation and cryo-EM analysis (see above), we, therefore, first simulated its protonation from ^ND1^Asp199, which is located within 6–7 Å. We find that the protonation of ^ND1^Asp199 is isoenergetic with the charge neutral state of ^ND1^Glu202, and the two states separated by an energy barrier (6–7 kcal/mol, Figure 6, left panel, see also Table S5, setups R1′, R2′). With an extra proton modeled on ^ND1^Glu204, the activation energy barrier reduces and protonated state of ^ND1^Glu202 is stabilized by ∼3 kcal/mol (Figure 6, left panel). Next, we proceeded with the simulation of transfer of proton from ^ND1^Glu202 to the location of the proton hole (i.e., ^ND1^Glu192). We note that this path is not observed in the cryo-EM structure, instead forms upon flipping of the ^ND1^Tyr142 side chain in MD simulations thereby leading to a connectivity involving 4–6 water molecules (Table S4, setups M1′, M2′, M4′, M5′) between the acidic residues (see Figure 6, lower right panel). When ^ND1^D199 is modeled charge neutral, a spontaneous proton transfer occurs from ^ND1^Glu202 to ^ND1^Glu192 within 4–5 ps. In case when ^ND1^D199 is anionic, the latter proton transfer is endergonic by 1–2 kcal/mol (Figure 6, right panel, see also Table S4, setups M5′). On the other hand, neutralization of ^ND1^Glu204 in TMH5-6 loop is found to yield favorable energetics of ^ND1^Glu202 → ^ND1^Glu192 proton (Figure 6, right panel, see also Table S4, setups M1′). In all cases of proton transfer from ^ND1^Glu202 to ^ND1^Glu192, the OE1 atom of the latter residue accepts proton from the neighboring water, whereas the proton donation to ^ND1^Glu143 occurs from its OE2 atom without any major conformational change of the carboxylate group (Figure S6).

**Figure 6:**
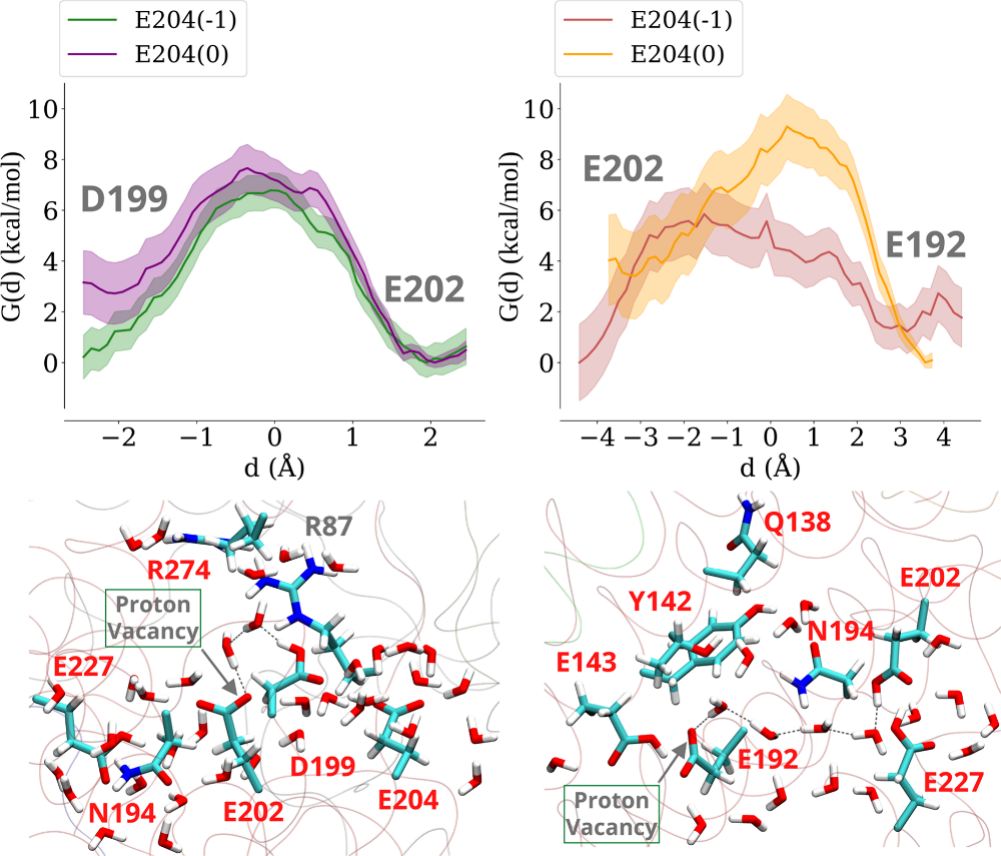
Protonation reactions in the Q tunnel/E channel junction. Proton transfer between ^ND1^Asp199 and ^ND1^Glu202 is isoenergetic and improves with charge changes in the vicinity (left panel; see text). Protonation of the proton hole at ^ND1^Glu192 from ^ND1^Glu202 under different conditions (right). The sufficiently hydrated QM regions and proton transfer routes explored are marked in lower panels, which display starting configurations used in QM/MM simulations. The shaded areas in energy profiles mark the statistical errors collected from the umbrella sampling windows (see Methods).

### Proton Transfer from the Membrane Domain toward the Q Tunnel/E Channel Junction

The mitochondrial complex I is a reversible enzyme such that under conditions of a high proton motive force or a higher [ubiquinol]/[ubiquinone] ratio, it operates in reverse and catalyzes the reduction of NAD^+^.^51^ In such a scenario, the proton transfer through the E channel may be reversed, occurring from the membrane domain toward the junction of the E channel and Q tunnel. Moreover, proton pumping models have been proposed in which proton transfer takes place toward the Q tunnel from the membrane domain.^21^ Considering the possibility that the E channel region may catalyze transfer of protons in the reverse direction, we analyzed the energetics of proton transfer reactions first at the E channel/Q tunnel junction starting from the p*K*_a_ calculation-based charge state. We find that the protonation of a proton hole on ^ND1^Glu202 from its neighbor ^ND1^Glu227 through a water molecule is unfavorable in the cryo-EM structural conformation. In an unbiased QM/MM MD simulation, proton modeled on ^ND1^Glu202 transfers to ^ND1^Glu227 within <1 ps (Figure S9). This result is in agreement with our p*K*_a_ predictions, which show that ^ND1^Glu202 is preferentially anionic in nature and ^ND1^Glu227 is charge neutral (Table S1). In an unbiased ∼ 5 ps QM/MM MD simulation initiated from the structural conformation observed in cryo-EM, we did not observe a proton transfer path between ^ND1^Glu192 and ^ND1^Glu202. Therefore, we probed our classical MD simulation trajectories, which revealed a path involving three-water molecules between ^ND1^Glu202 and ^ND1^Glu192 (Figure S10). It is noteworthy that this path is established in cases when ^ND1^Tyr142 flip occurs. When we analyzed the protonation of ^ND1^Glu202 from ^ND1^Glu192 (by passing ^ND1^Glu227), the charge neutral states of both residues are found to be nearly isoenergetic (Figure 6). Importantly, in the state in which ^ND1^Glu192 is anionic, it does not accept a proton from ^ND1^Glu143. When energetics of the latter proton transfer step is evaluated with QM/MM free energy simulations, it is found that the protonation of ^ND1^Glu192 is unfavorable (Figure S9). These data indicate that proton transfer in the reverse direction suffers from energetic bottlenecks. However, with the additional negative charges in the region created upon quinone reduction during enzymatic turnover or due to its migration in the tunnel, the energetics can become favorable. We note that since several acidic residues in TMH5-6 region are modeled anionic (see Table S1), there is an inherent thermodynamic drive for protons toward the latter section. For instance, the proton transfer from ^ND3^Glu34 to ^ND4L^Asp66 (Figure 2) is energetically favorable in a charge state derived from the p*K*_a_ calculations. Overall, our results indicate that transfer of protons through the E channel can also occur in the opposite direction with charge changes on either side of it.

## Discussion

Proton transfer reactions in proteins are catalyzed by water molecules and side chains of polar amino acid residues. Such reactions can be studied in a quantum mechanical framework,^8,10,52^ also involving the methods such as DFTB^53^ or EVB.^54,55^ In addition to the high spatial and temporal resolution of protonation dynamics provided by these approaches, they can also be used to evaluate the energetics of proton transfer. Even though large-scale conformational changes are not easily captured by computationally demanding DFT-based QM or QM/MM MD simulations, 3D coordinates or structural ensembles of model systems are usually obtained from high-resolution X-ray and cryo-EM and/or from classical MD simulations, providing starting points to initiate proton transfer reactions on a classical potential energy surface.

Here, we studied one well hydrated putative proton transfer pathway (E channel) in a high-resolution structure of respiratory complex I,^16^ which is a redox-driven proton pump in the bioenergetic membranes of mitochondria and several bacteria.^56^ Because of its channel-like architecture, the presence of several conserved acidic residues and extensive hydration, the E channel in complex I has been proposed to catalyze redox-driven proton transfer.^22,25,27^ Several site-directed mutagenesis studies have also revealed the importance of titratable residues in the E channel in redox-coupled proton pumping activity of complex I.^57–61^ In this work, we show that proton transfer through the E channel of complex I can occur with low activation energy barriers. The highest energy barriers obtained for thermodynamically favorable proton transfer reactions (Δ*G* ≤ 0) are 6–7 kcal/mol (*k* ∼ 1–10 μs, with A ∼ 10^10^ s^–1^), which is reasonable considering the millisecond time scale of complex I turnover.

It is well-known that long-range electrostatics play a central role in enzyme catalysis.^62–64^ We also find that long-range effects are important in enhancing both the exergonicity and kinetics of the proton transfer process. Not only we see that a unit charge change (±1e) in the buried low dielectric (MM) region of the protein rearranges water chain, but it also affects the proton transfer energetics in the QM region situated at up to 15–20 Å apart. This in turn means that for complex I, the redox reactions in the Q tunnel (or its vicinity) can drive proton transfer in the membrane arm of complex I commensurate with the long-range electron–proton coupling seen in this enzyme.^19,22^ We find that a proton on the proposed proton loading site region (^ND1^Asp199)^17,19^ at the junction of membrane and hydrophilic arms of complex I can drive proton transfer in the E channel region and beyond. Since large-scale conformational transitions are not accounted for in the hybrid QM/MM MD simulations, such changes in energetics are likely to originate due to the long-range nature of electrostatics.

A recent QM/MM study explored the energetics of transfer of a proton on a water path involving 4–5 water molecules between ^ND4L^Glu34 and ^ND3^Asp66.^10^ The data revealed proton transfer to be endergonic by ∼5 kcal/mol with activation energy barriers around 10 kcal/mol. Earlier, using mammalian mitochondrial complex I structural data and classical and QM/MM MD simulations energy barriers of ∼15 kcal/mol were reported for the same proton transfer reaction in the active (A) state of the enzyme.^28^ In contrast, our QM/MM simulations on the same but shorter path involving three-water molecules show much more favorable energetics. We think this is in part related to the selected charge states of the residues in the QM and MM regions, especially the TMH5-6 loop, which can perturb the energetics of proton transfer (see above).

Several high-resolution cryo-EM structures of respiratory complex I have been resolved in different catalytic states, namely, open/closed or deactive/active.^21,42^ The highly conserved ^ND1^Tyr142 is seen to occupy two different conformational states in the cryo-EM structures. In our classical MD simulations initiated from the active (resting) state, we observed conformational dynamics of tyrosine, which in its flipped conformation allows a direct hydrogen bonding interaction between the two acidic residues ^ND1^Glu192 and ^ND1^Glu143 of the E channel. This interaction is found to be important for proton transfer in the E channel, which would not occur in the presence of intervening tyrosine, because of its unfavorable anionic state (high proton affinity). This is in contrast to earlier study where conserved tyrosine residues in the central hydrophilic axis of the ND2 subunit of complex I were found to undergo protonation/deprotonation reactions.^8^ We suggest that the conserved tyrosine residue in the ND1 subunit acts as a gating element and likely prevents the flow of protons between the E channel/Q tunnel region and the extended membrane domain of complex I. The conformational flipping of tyrosine allows for proton transfer to occur through this route during turnover, which drives the proton pump of complex I.

Analysis of the energetics of proton transfer in several subsections of the E channel region allows us to combine the free energy profiles and propose a molecular mechanism of proton transfer in the E channel region (Figure 7). We start from a scenario when a proton vacancy is created at the end of the E channel region (i.e., on ^ND3^Asp66) upon proton pumping to the P side via extended membrane domain of the complex. Our QM/MM free energy calculation results show that protonation of a single TMH5-6 loop residue (^ND1^Asp199) is sufficient to initiate an energetically favorable cascade of proton transfers across the entire breadth of the E channel: from ^ND1^Asp199 all the way to ^ND3^Asp66. Noteworthy, spontaneous proton transfer from ^ND1^Glu143 to ^ND3^Asp66 should be followed by a conformational flip of tyrosine ^ND1^Tyr142, which opens a direct proton pathway between ^ND1^Glu192 and ^ND1^Glu143. Finally, protonic equilibration takes place in the TMH5-6 loop region between protonated ^ND1^Asp199 and anionic ^ND1^Glu202. This is followed by the transfer of the proton from the latter to ^ND1^Glu192 via a water wire.

**Figure 7:**
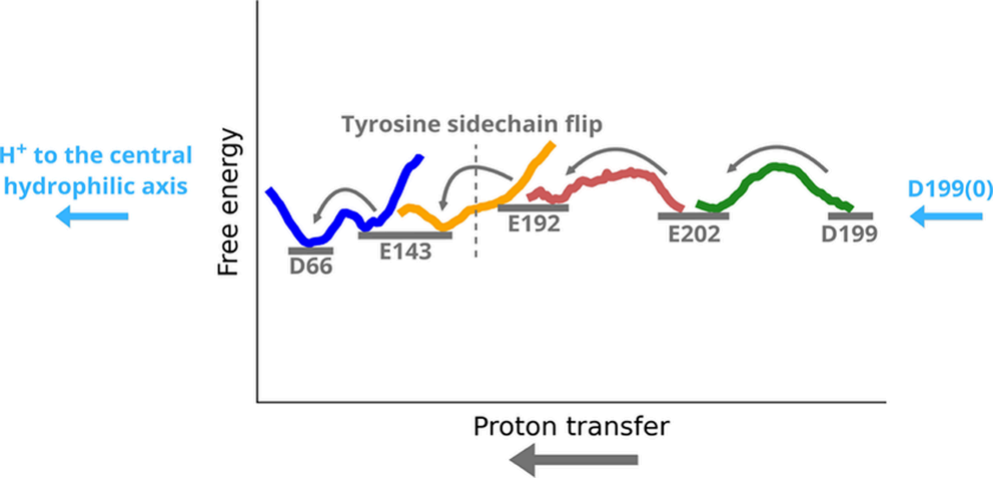
A stepping-stone mechanistic model showing that the protonation of a single residue (i.e., ^ND1^Asp199) is able to drive the overall reaction of proton transfer through the E channel region (shown from right to left). The free energy profiles describe the proton transfers between the marked protein residues and correspond to the following simulation setups: R1′ (green), M5′ (red), M3′ (orange), L8 (blue). See also Tables S2, S4, and S5.

The proton transfer reactions modeled in our study are buried in the protein matrix insulated from the solvent environment (Figure 1). However, the transfer of a proton from ^ND1^Glu192 to ^ND4L^Glu34 in the E channel region occurs at an angle to the membrane plane. This roughly corresponds to a net shift of an elementary protonic charge by 5–10 Å in the direction of the membrane normal toward the positive side of the membrane. Under energized conditions with a proton motive force of ca. 180 mV across an ∼35 Å membrane, the proton transfer reaction will have a nominal energy cost of around 20–50 meV (∼1 kcal/mol), which is likely to be compensated in part by its progression energetically downhill (see overall trend in Figure 7). Overall, the proton transfer through the E channel region may not incur major costs on the net free energy available from ubiquinone reduction by NADH (ca. 800 meV), thereby not endangering the key function of the enzyme, i.e., proton uptake and release from the N side to the P side of the membrane.

The directionality of proton transfer in the E channel may depend on if complex I functions in the forward mode catalyzing redox-coupled proton pumping or in reverse mode in which NAD^+^ is reduced from ubiquinol.^51^ The proton transfer direction is not known in either of the two scenarios, but our results suggest that protons can flow through the E channel with low activation energy barriers. The protonation of proton loading site in the TMH5-6 region enhances the driving force for a proton diffusion toward the extended membrane domain of complex I. On the other hand, anionic charges built up in the region upon quinone reduction can drive the transfer of protons also in the opposite direction. All in all, a high level of hydration of the E channel and presence of several acidic residues responds to the subtle change in charges on either side of the E channel triggering proton transfer in one or the other direction.

It has been proposed that quinone binding, its dynamics as well as its reduction,^65–69^ can trigger conformational transitions in the vicinity of the Q tunnel, including the E channel region. Even though a quinone molecule is not modeled in the cryo-EM structure studied here, it can be envisaged that the protonation of anionic intermediates of quinone like semiquinone or quinol^17,68^ and/or amino acid residues can alter proton transfer energetics in the E channel.^10,19,21,23^ Such effects will be more pronounced in case the negative charges build up during the quinone reduction are closer to the E channel/Q tunnel junction, such as the TMH5-6 region.

The origin of the proton traveling through the E channel is another challenging issue; is it delivered by doubly reduced/doubly protonated quinol upon its oxidation^29,70^ or its deprotonation^10^ or is the proton taken up from the N phase of the membrane as part of the quinone redox chemistry? ^17,19,50^ Several different routes have been proposed based on structures and computer simulations that can transfer protons from the bulk N phase to the protein interior. Parey, Lasham et al.^17^ and Yoga et al.^80^ have highlighted the importance of protonatable residues from NDUFS2 and ND3 subunits, respectively, in proton transfers. Similarly, high-resolution structures have revealed hydrated paths that can deliver protons to the region of Q tunnel and its surroundings.^15,42,71^ Irrespective of the source and destination of the proton, the data suggest that proton can flow through the E channel region without major energetic bottlenecks (flipped tyrosine) under optimal charge and conformational conditions.

## Methods

We performed hybrid QM/MM and fully atomistic classical MD simulations on a cryo-EM structure of mitochondrial respiratory complex I from*Mus musculus* (PDB ID: 8OM1).^16^ We used the mouse active-resting-state structure of complex I as the basis for QM/MM calculations because of its high-resolution (∼2.4 Å) and the high level of hydration observed in the E channel region. The structure displays well-connected putative proton transfer paths formed by structurally resolved water molecules bridging the polar and charged amino acid residue side chains (see Figure 1). The pre-existing paths in a high-resolution structure set the stage for a direct investigation with QM/MM approaches, without involving the extensive usage of classical MD simulations, which can indeed be utilized to induce structural transitions and enhance hydration but can also lead to alternative paths and force field and charge-dependent biases. However, at the same time, it is also well-known that the results of QM/MM modeling, among other factors, depend on the protein conformation chosen. Since hybrid QM/MM MD simulations are relatively slow and cannot encompass the desired structural conformations, fully atomistic classical MD simulations were employed to sample the conformational space and hydration in those areas where proton transfer paths were unclear (e.g., route between ^ND3^Asp66 and ^ND4L^Glu34 or between ^ND1^Glu192 and ^ND1^Glu143, see Results). Classical MD simulations were carried out with NAMD 2.14,^72^ while QM/MM MD simulations were performed with NAMD 2.14 ^73^ coupled to ORCA 5.0.3.^74,75^

For the model system, we included the seven core subunits of respiratory complex I along with their corresponding structurally resolved water molecules (Figure 1). The selected subunits are NDUFS2 (49 kDa), NDUFS7 (PSST), ND1, ND3, ND6, ND4L, and ND2. The N2 iron–sulfur cluster from the PSST subunit was also incorporated in its reduced form. Residues 1–39 of the 49 kDa subunit form a highly flexible and disordered terminus and were excluded from the model system. Unresolved hydrogen atoms, along with the water box (TIP3P water model^76^), and ions (150 mM Na^+^/Cl^–^ ^77^) were added using VMD PSFGEN plugin.^78^ The initial protonation states of amino acids were assigned based on empirical p*K*_a_ predictions from PROPKA software.^35,79^ The membrane lipid bilayer around the protein comprised classically equilibrated POPC (54%), POPE (34%), and cardiolipin (12%). Overall, our model system comprised ∼220,700 atoms. We note that truncated model systems of respiratory complex I have successfully been simulated in the past for long time scales and were found to be stable and provided functional insights.^29,68,80^

The accurate description of the charge state of the protein is a challenging issue. Several computational methods exist to identify the protonation states of amino acid residues in a given conformation and environment of the protein.^81^ The rapidly evolving constant pH MD simulation methods^82,83^ can be utilized to identify the protonation states of amino acid residues. However, some challenges remain as recently reviewed.^84^ The fast p*K*_a_ prediction software Propka,^79^ which is utilized here, yields reasonable proton affinity estimates, especially for buried residues^39^ (see also refs 40 and 41). The cryo-EM density maps can also provide information on the charged states of amino acid residues.^85^ Previously, we have analyzed the cryo-EM map of respiratory complex I and suggested that some of the truncated densities may represent anionic (charged) states of acidic amino acid residues in the E channel and TMH5-6 regions, whereas well-resolved regions may correspond to charge neutral (or also anionic, depending on the surroundings) states of acidic residues.^35,36^ In the cryo-EM map of complex I structure utilized in this study,^16^ weaker densities of residues ^ND1^Glu202 and ^ND1^Glu204 are observed, which have been modeled as anionic (Table S1). In contrast, the maps of ^ND1^Glu192 and ^ND1^Glu143 are relatively well-resolved, and they have been modeled as charge neutral (Table S1).

To observe proton transfer dynamics and assess its energetics, we carried out unbiased and biased hybrid QM/MM MD simulations of the E channel and its surrounding regions. The entire E channel region was partitioned into separate QM subregions (see Figure S1, also Tables S2–S6), and all relevant water molecules were included. The unbiased QM/MM simulation protocol commenced with a classical energy minimization using the conjugate gradient (CG) algorithm, with positional restraints (99 kcal mol^–1^ Å^–2^) applied to the heavy atoms of the protein and the metal centers as well as the oxygen atoms of water molecules. This was followed by a 10 ns classical MD simulation (equilibration) with the same restraints. Next, a 200-step CG QM/MM minimization without restraints was carried out, followed by a 5 ps QM/MM MD production run. For free energy calculations, we used the umbrella sampling (US) method,^86^ where the reaction coordinate/collective variable for our US simulations was constructed using the Colvars module,^87,88^ employing a linear combination of interatomic distances along the proton transfer pathway:

$$d=\left(d_{1}-d_{2}\right)+\left(d_{3}-d_{4}\right)+\left(d_{5}-d_{6}\right)$$

See also Figure S11.

Using the harmonic biasing potential,

$$U\left(d\right)=(1/2)\;k{\left(d-d_{0}\right)}^{2}$$

we restrained the reaction coordinate to its initial value (*d*_0_) in the zeroth simulation window. The force constant (*k*) was set to 100 kcal mol^–1^ Å^–2^. Subsequent windows were obtained by incrementally pulling *d*_0_ by 0.2 Å in both directions. This was followed by a 1.8 ps equilibration and finally a 2 ps production run. The potential of mean force (PMF) profiles were obtained with a weighted histogram analysis method with the WHAM analysis tool.^89^ The error bars were computed using the Monte Carlo bootstrapping technique with 1000 trials and 30–50 fs correlation time. The convergence of free energy profile and overlap of biased histograms is displayed in Figure S12 for selected simulation setups.

The fully atomistic classical MD simulations commenced with an energy minimization using the CG algorithm with harmonic position restraints (99 kcal mol^–1^ Å^–2^) to protein and cofactor heavy atoms and oxygen atoms of structurally resolved water molecules. This was followed by a 10 ns classical equilibration with the same restraints, a subsequent 10 ns equilibration with restraints on the protein Cα atoms, and finally a 0.5 μs production run. Three independent simulation replicas were initiated to enhance statistics. Since the breakage of the water wire between ^ND3^Asp66 and ^ND4L^Glu34 was observed in QM/MM MD simulations (see setup L3, Table S2), we also performed an additional ∼0.5 μs classical MD simulation with the identical protonation states to test whether this disruptive effect is retained under classical treatment.

All of our simulations were set up in the isothermal–isobaric (NPT) ensemble at a temperature of 310 K and a pressure of 1 atm. The conditions were attained using Langevin thermostat^90^ and Langevin piston barostat.^91,92^ Trajectory was propagated via Verlet integration scheme with a 1 fs time step. Nonbonded interactions for MM atoms were handled with the Verlet^93^ cutoff algorithm with a 12 Å cutoff, a 10 Å switching distance, and a 14 Å pair list distance. CHARMM^94^ force field was used to define the MM framework. The QM regions in the simulations were handled using the B3LYP^95–97^ density functional with def2-SVP basis set,^98^ supplemented by DFT-D3 dispersion correction to account for long-range London dispersion forces.^99^ Similar DFT-based theoretical models were found to adequately describe the proton transfer reactions in model systems (see e.g., refs 100 and 101) and in proteins with good agreement with experimental data.^102,103^ Also, based on our own previous studies of similar systems,^8,68^ we note that changing the DFT functional/basis set does not significantly alter the free energy profiles. A tolerance of 1.0 × 10^–8^ au was used for SCF energy convergence. The QM-MM interface interaction was handled with additive coupling and electrostatic embedding scheme.^73^

## Supplementary Material



## Data Availability

The 3D coordinates of the structure of respiratory complex I (PDB id 8OM1) are available from the PDB (https://www.rcsb.org/). NAMD (https://www.ks.uiuc.edu/Research/namd/), ORCA (https://www.faccts.de/orca/), VMD (https://www.ks.uiuc.edu/Research/vmd/), colvars module (https://colvars.github.io/master/colvars-refman-namd.html) and Propka (https://github.com/jensengroup/propka) software are available for download from their respective webpages. Standard CHARMM force field files are available from CHARMM developers (see references in the Methods section). Data required to perform all classical and QM/MM MD simulations is available at https://doi.org/10.5281/zenodo.15747940.

## Notes

Supplementary Tables S1–S6 and Figures S1–S12; Table S1 – Calculated p*K*_a_ of amino acid residues; Table S2 – QM/MM simulation setup describing L region; Table S3 – QM/MM simulation setup describing M region; Table S4 – QM/MM simulation setup describing M′ region; Table S5 – QM/MM simulation setup describing R′ region; Table S6 – QM/MM simulation setup describing R region; Figure S1 – QM regions studied in this work; Figure S2 – Formation of a water wire between ND3Asp66 and ND4LGlu34; Figure S3 – Effect of length of water wire on proton transfer energetics; Figure S4 – Long-range effects on the conformational dynamics of water molecules upon charge change; Figure S5 – QM/MM-based relaxation abridges the structural gap; Figure S6 – Proton transfer through an acidic residue involves different oxygen atoms of its carboxylate group; Figure S7 – Energetics of proton transfer bypassing the conserved tyrosine; Figure S8 – Conformational dynamics of tyrosine; Figure S9 – Protonation dynamics and energetics in R and M′ regions; Figure S10 – Formation of a water wire between ND1Glu202 and ND1Glu192 in classical MD simulations; Figure S11 – Reaction coordinate applied in QM/MM free energy simulations; Figure S12 – Convergence of free energy (*G*(*d*)) profiles and overlap of umbrella sampling histograms (PDF)
