# Supplementary material for: Proton Transfer through a Charged Conduit in Respiratory Complex I: Long-Range Effects and Conformational Gating
Source: J Chem Inf Model. 2025 Sep 18;65(19):10600–12. doi: 10.1021/acs.jcim.5c01365 (PMC12529768; doi:10.1021/acs.jcim.5c01365)
Supplement: Supplementary file 1 [file ci5c01365_si_001.pdf]

Supplementary Information

Proton transfer through a charged conduit in respiratory complex I – long range effects and conformational gating

Luka Simsive[1], Oleksii Zdorevskyi[1,*], Vivek Sharma[1,2,*]

[1]Department of Physics, University of Helsinki, Helsinki, Finland

[2]HiLIFE Institute of Biotechnology, University of Helsinki, Helsinki, Finland

Correspondence to;

Oleksii Zdorevskyi (oleksii.zdorevskyi@helsinki.fi)

Vivek Sharma (vivek.sharma@helsinki.fi)

Residue	Calculated pK_a*	Sidechain charge†
^{ND1}D199	3.29	-1
^{ND1}E202	5.44	-1
^{ND1}E204	6.92	-1
^{ND1}E206	4.11	-1
^{ND1}E143	7.21	0
^{ND1}E192	7.86	0
^{ND1}E227	7.96	0
^{ND1}E262	7.80	0
^{ND1}H93	7.08	+1
^{ND1}R274	15.89	+1
^{ND3}D66	8.05	0
^{ND3}E68	8.56	0
^{ND3}E105	7.89	0
^{ND6}E127	7.75	0
^{ND4L}E34	7.62	0
^{ND4L}E70	9.73	0
^{ND4L}H25	9.68	+1
^{ND2}K135	6.92	0
^{NDUFS2}D104	11.30	0
^{NDUFS2}D160	8.30	0
^{NDUFS2}E343	8.45	0
^{NDUFS2}E427	8.60	0
^{NDUFS2}H409	7.11	+1
^{NDUFS7}R87	11.19	+1

Table S1. Calculated pK_a of amino acid residues and their modelled charge state.

* pK_a is calculated using Propka tool [1] on the high-resolution structure of complex I (PDBID: 8OM1 [2]).
† Alternative charge states of amino acid residue have been modelled (see text). Charge state of the residue can change if the residue is in the QM region (but remains fixed in the MM region).

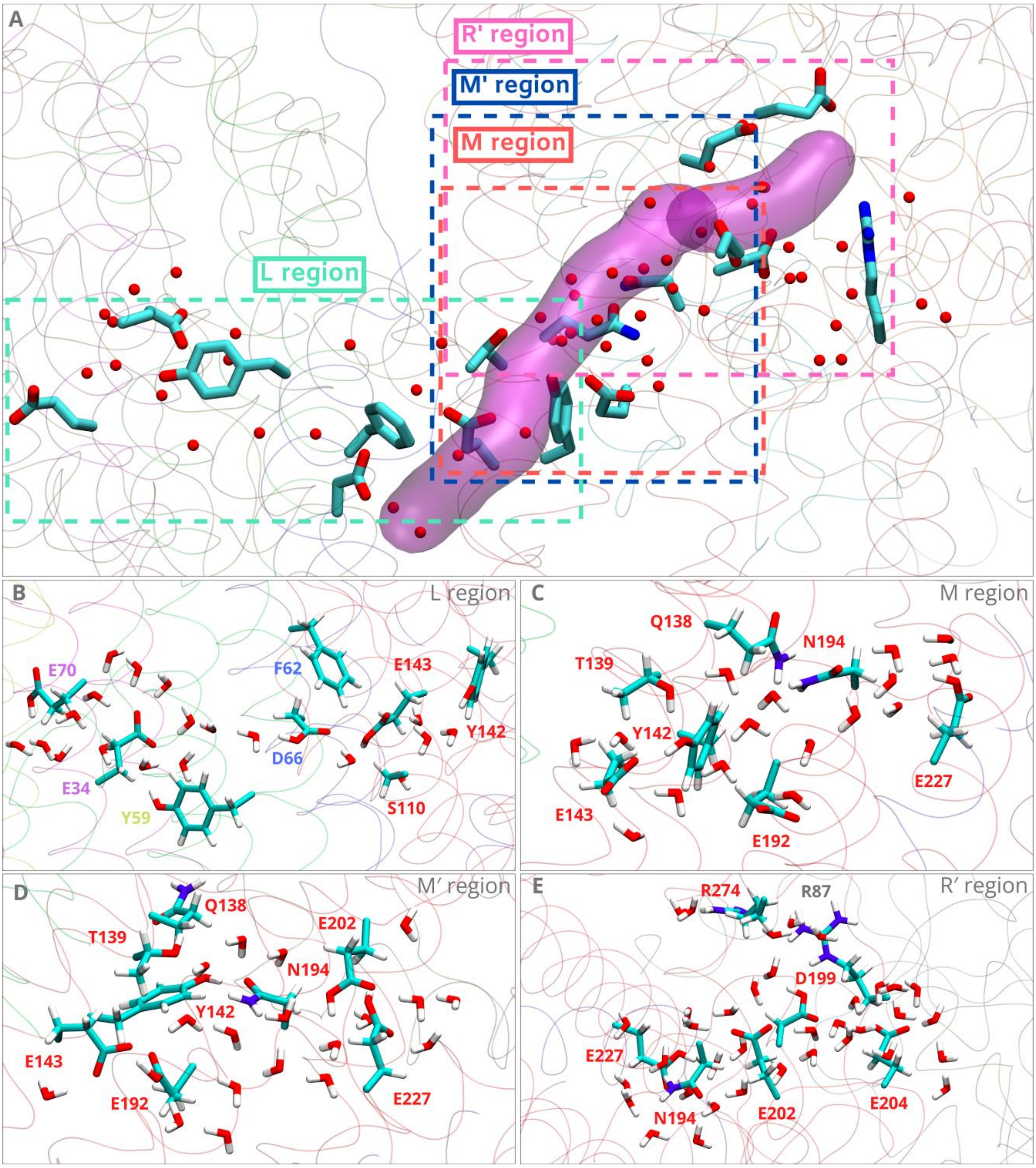

Figure S1. QM regions studied in this work. (A) The E channel region is shown with several QM subregions studied in this work marked. (B) L region, spanning the interface between ND1, ND3, and ND4L subunits. (C) M region connecting the L region with the central part of the ND1 subunit. (D, E) M′ and R′ regions created from the MD simulation snapshot where ^{ND1}Tyr142 was "flipped" from its structural conformation (see main text, "Conserved tyrosine as a conformational gate"). Protein residues and water molecules belonging to the QM region are shown in licorice. Amino acids labels are colored according to the subunit they belong to: ND1 (red), ND3 (blue), NDUFS7 (grey), ND6 (green), and ND4L (purple).

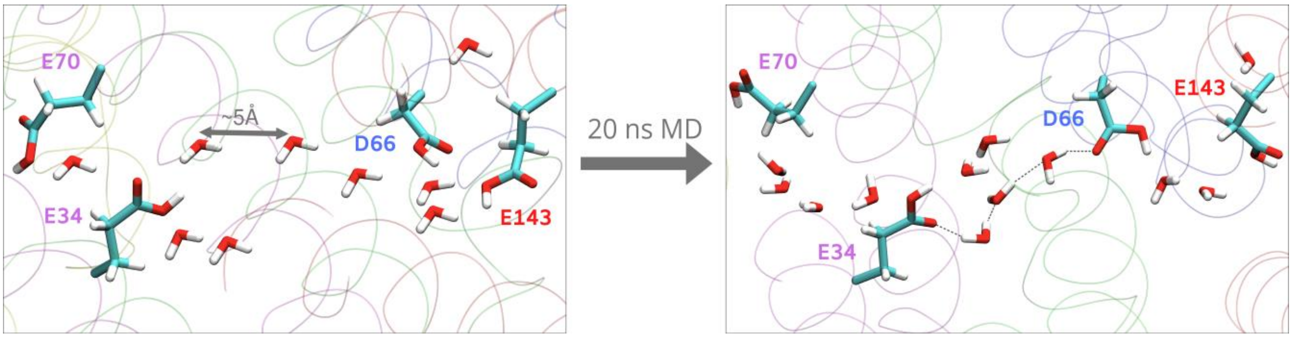

Figure S2. Formation of a water wire between ^{ND3}Asp66 and ^{ND4L}Glu34 (see methods). (Left) The high-resolution structure of complex I (PDBID: 8OM1 [2]) shows a break in the water-based hydrogen-bond connectivity between ^{ND3}Asp66 and ^{ND4L}Glu34 due to a ~5 Å gap (oxygen-oxygen distance), which is abridged upon a 20-ns classical MD equilibration with constraints on protein backbone atoms (right, see also methods). Labels of the protein residues are coloured according to the respective subunits: ND1 (red), ND6 (blue), and ND4L (purple).

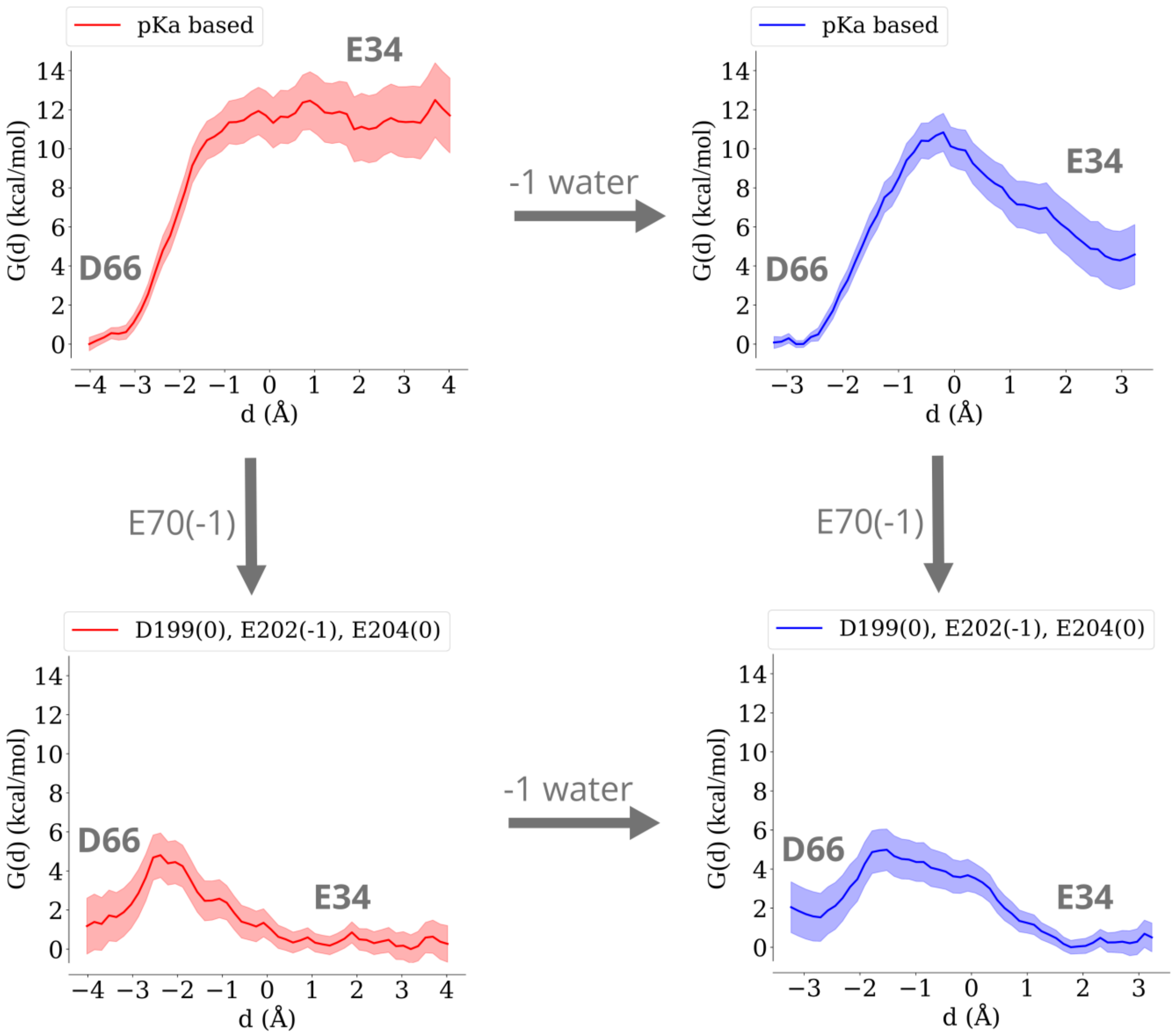

Figure S3. Effect of length of water wire on proton transfer energetics. Proton transfer energetics between ^{ND3}Asp66 and ^{ND4L}Glu34 changes with when proton transfer wire between them consists of four (left panel) and three (right panel) water molecules. The bootstrapping errors (see methods) are shown as shaded areas around the respective free energy profiles.

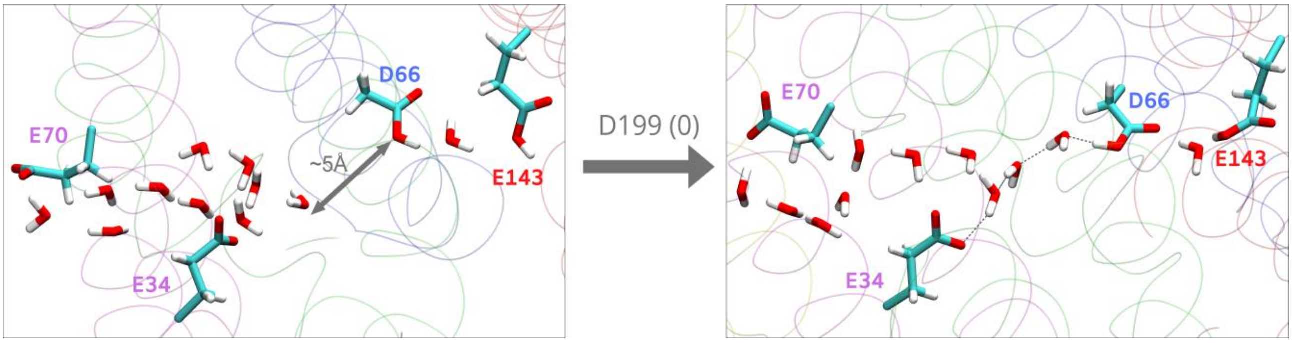

Figure S4. Long range effects on the conformational dynamics of water molecules upon charge change. QM/MM MD simulation snapshot shows the disruption of the water wire (gap of ~5 Å, oxygen-oxygen distance) between ^{ND3}Asp66 and ^{ND4L}Glu34 when ^{ND4L}Glu70 is modelled anionic (left panel). However, the hydrogen bonding network is stable, when ^{ND1}Asp199, located ~20 Å from the region shown, is modelled charge neutral (^{ND1}Asp199 is not shown, but see Figs. 1 and 2). Labels of the protein residues are coloured: ND1 (red), ND3 (blue), ND6 (green) and ND4L (purple).

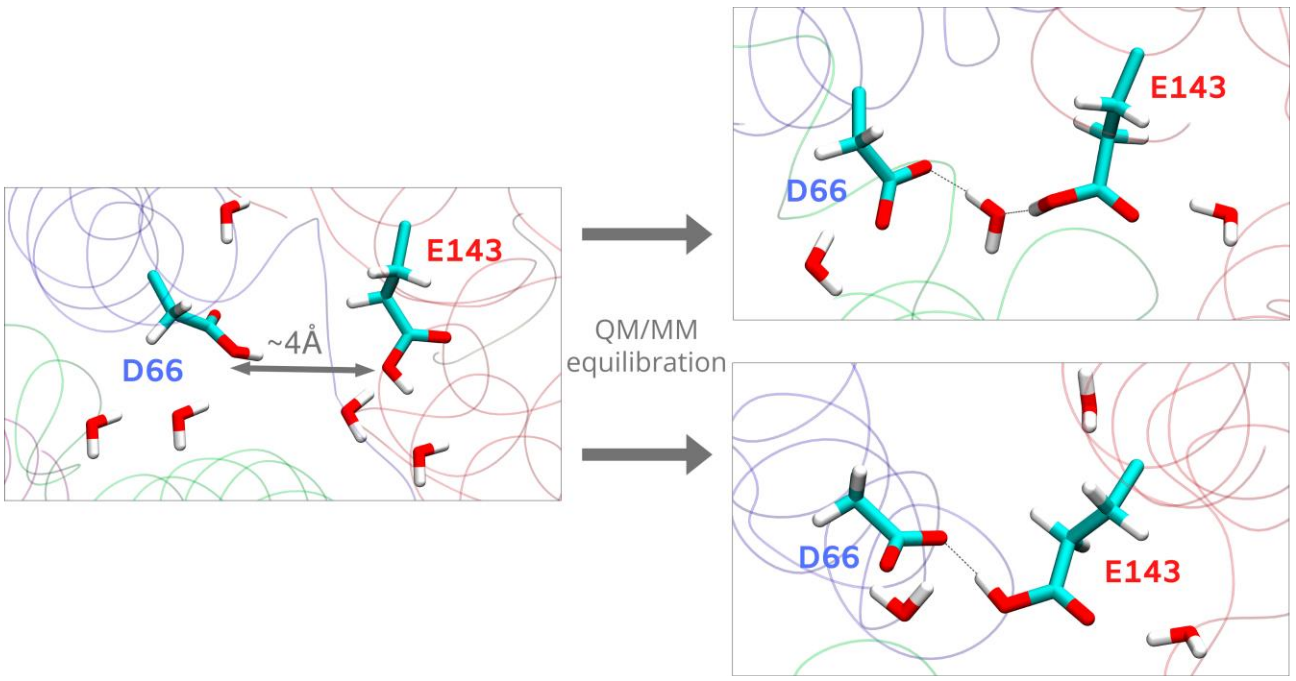

Figure S5. QM/MM-based relaxation abridges the structural gap. Formation of a hydrogen bond network between ^{ND1}Glu143 and ^{ND3}Asp66 during an unbiased QM/MM MD simulation (right), compared to the arrangement observed in the cryo-EM structure (left; PDBID: 8OM1 [2]).

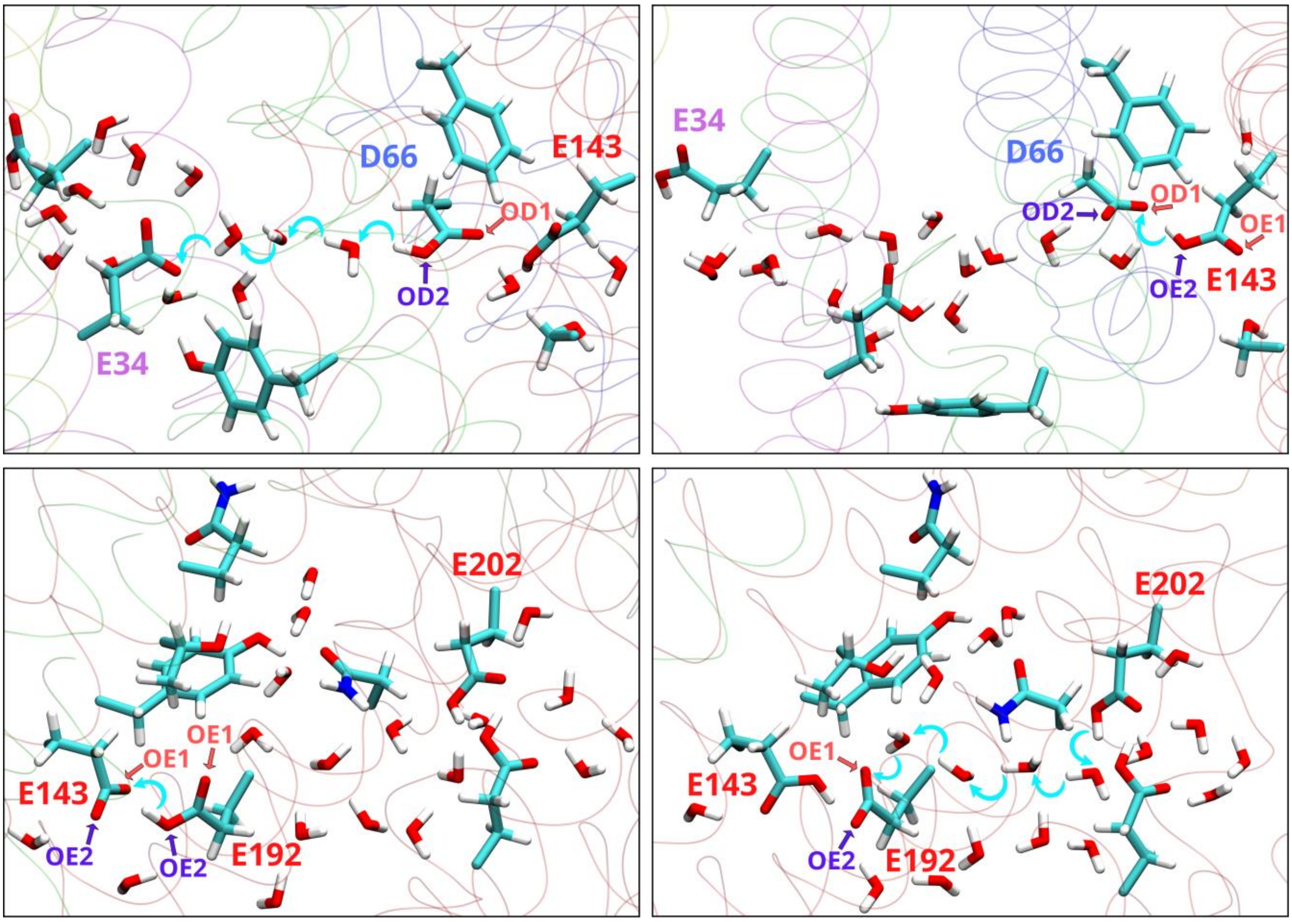

Figure S6. Proton transfer through an acidic residue involves different oxygen atoms of its carboxylate group. The proton bound to the OD2 or OE2 atom of ^{ND3}Asp66 or ^{ND1}Glu192 is delivered towards ^{ND4L}Glu34 or ^{ND1}Glu143, respectively. On the other hand, the OD1 or OE1 atom of ^{ND3}Asp66 or ^{ND1}Glu192 accepts protons from the neighbouring water molecules or amino acid residues. The proton transfers are marked with cyan colored arrows. OD1/2 and OE1/2 atoms corresponding to the oxygen atoms at delta(δ)/epsilon (ε) positions of aspartic/glutamic acid sidechains are marked in red and blue colors, respectively.

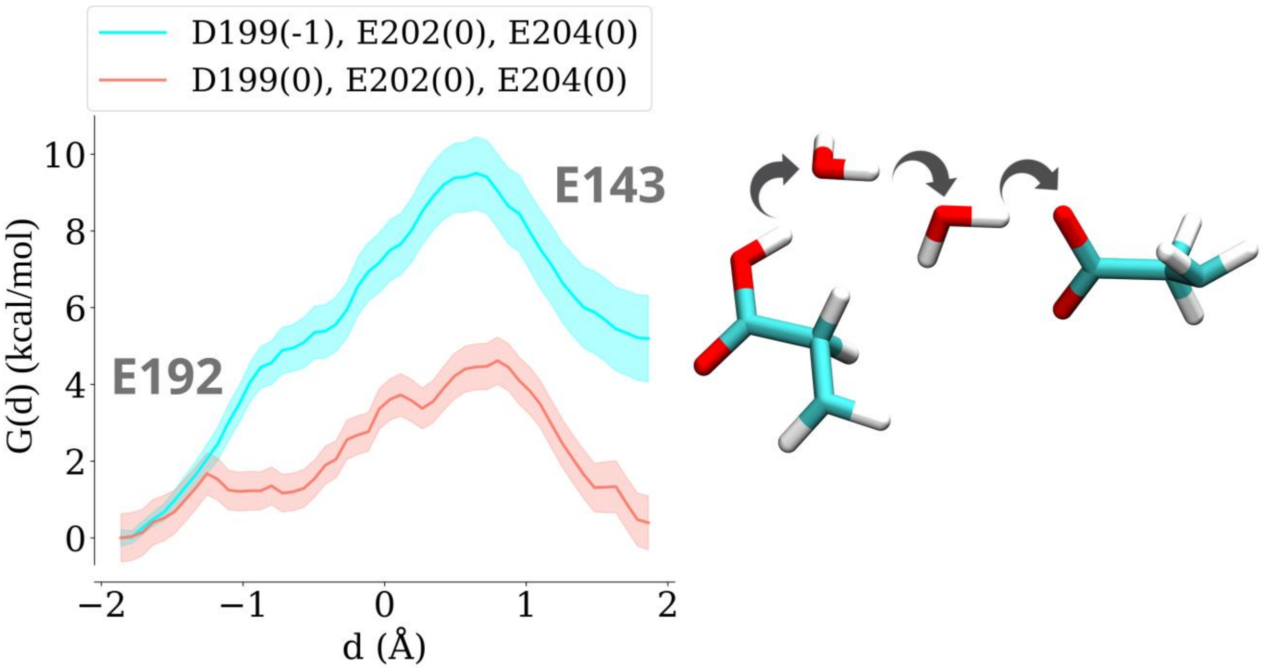

Figure S7. Energetics of proton transfer by-passing the conserved tyrosine. Potential of mean force (G(d), free energy) profiles for proton transfer from ^{ND1}Glu192 (left) to ^{ND1}Glu143 (Table S2, setups M10, M11) through the chain of 2 water molecules derived from QM/MM free energy calculations (left). Different colours represent different protonation states of the titratable residues. The studied proton transfer path via water molecules is shown on the right.

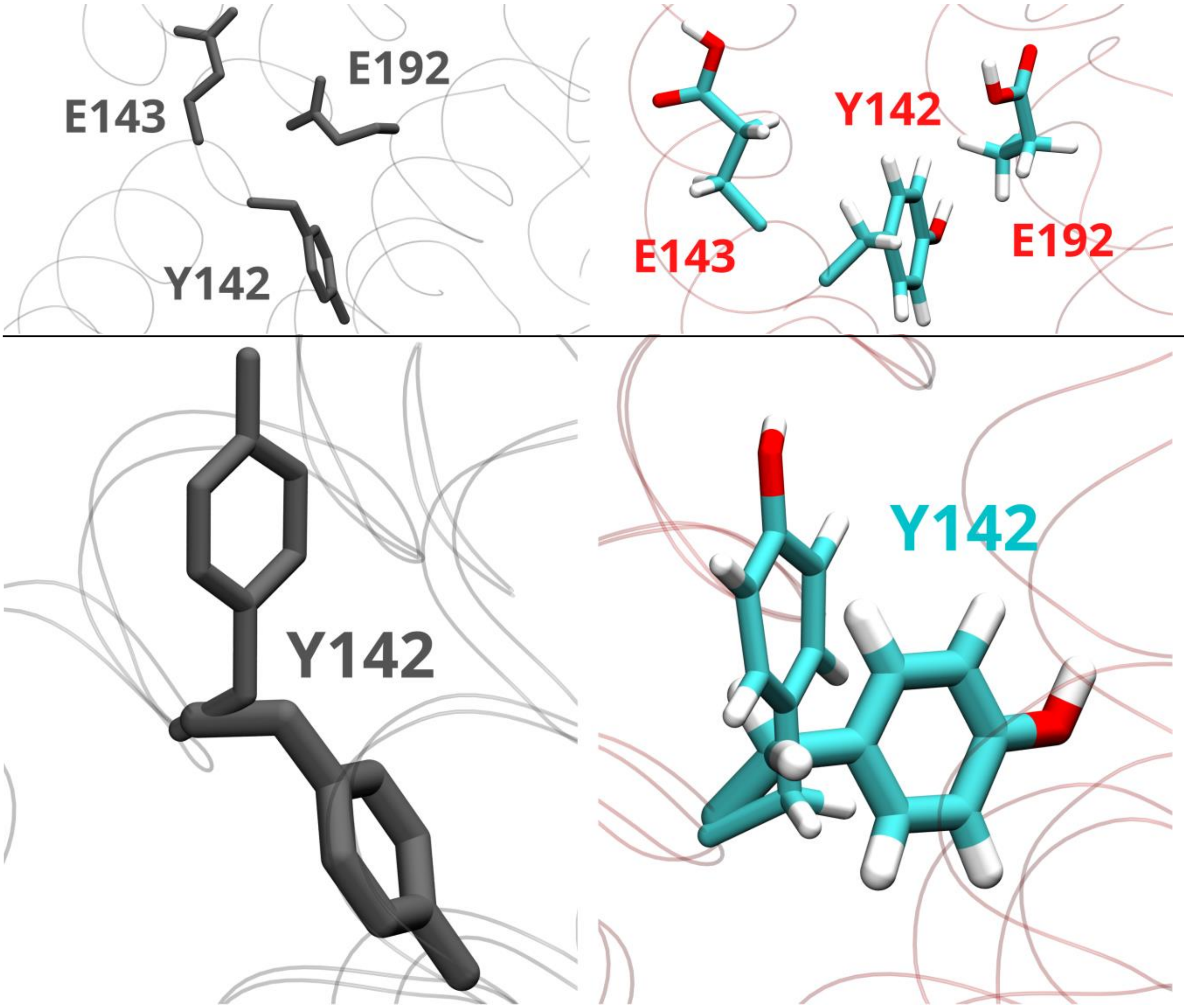

Figure S8. Conformational dynamics of tyrosine (^{ND1}Tyr142) in structures (grey) and simulations (cyan). Top panel - the flipped conformation of tyrosine and alternative conformations of neighbouring glutamic acids (^{ND1}Glu143 and ^{ND1}Glu192), see also Fig. 5. Lower panel – overlay of two conformations of tyrosine, with flipped conformations shown in thick licorice. The residues in grey color correspond to the conformations from cryo-EM structures PDB ids 6ZKA and 6ZKB.

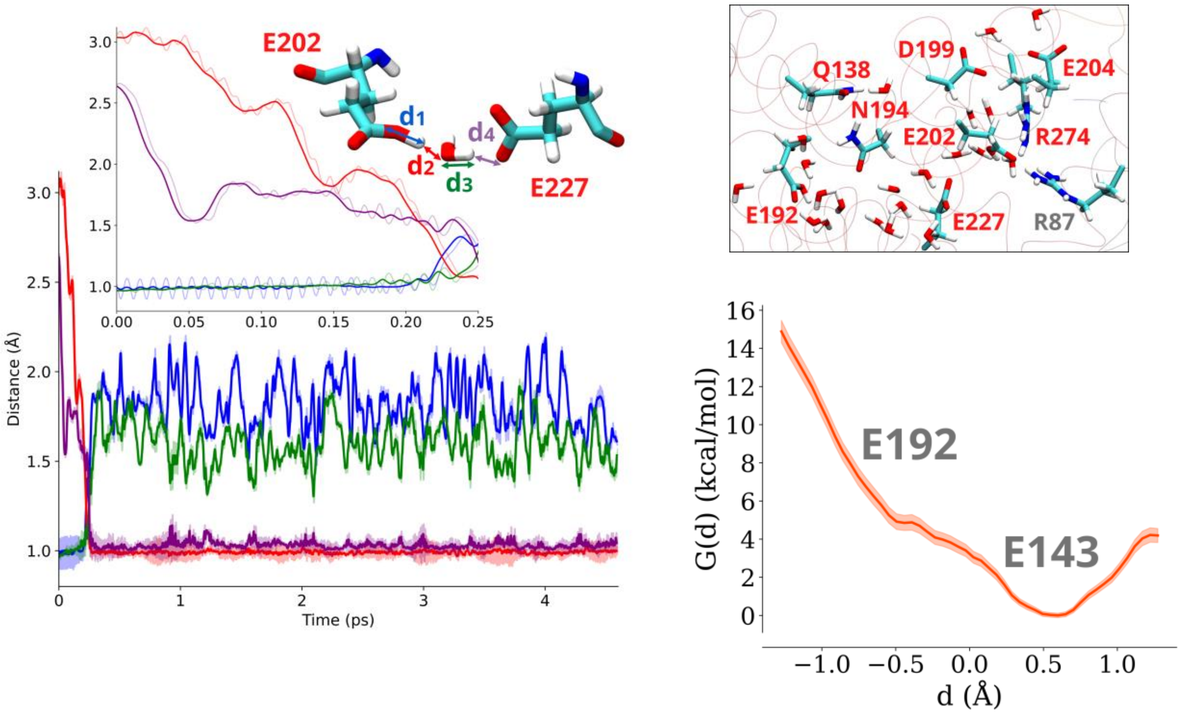

Figure S9. Protonation dynamics and energetics. (Left) Proton on ^{ND1}Glu202 is transferred to ^{ND1}Glu227 in an unbiased QM/MM MD simulation. The distances d_1-d_4 are plotted and marked. (Right, top) The QM region corresponding to protonation reaction studied between ^{ND1}Glu202 and ^{ND1}Glu227. (Right, lower) Free energy profile of proton transfer between ^{ND1}Glu143 and ^{ND1}Glu192.

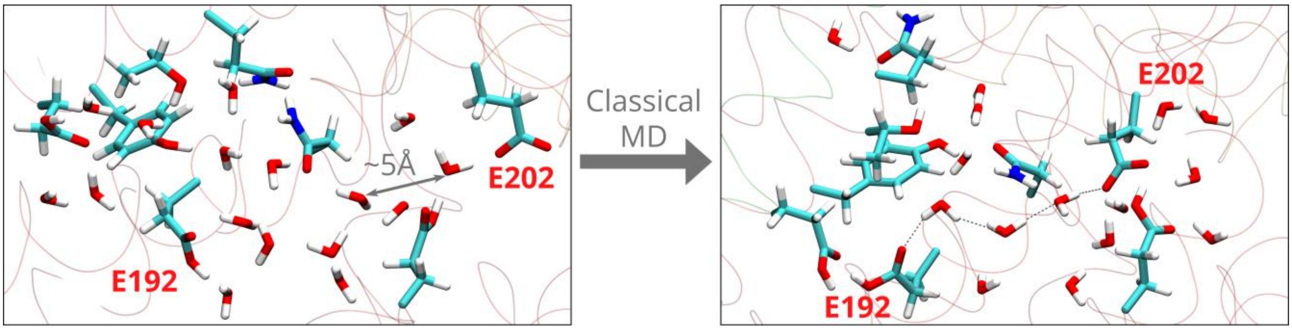

Figure S10. Formation of a water wire between ^{ND1}Glu202 and ^{ND1}Glu192 in classical MD simulations.

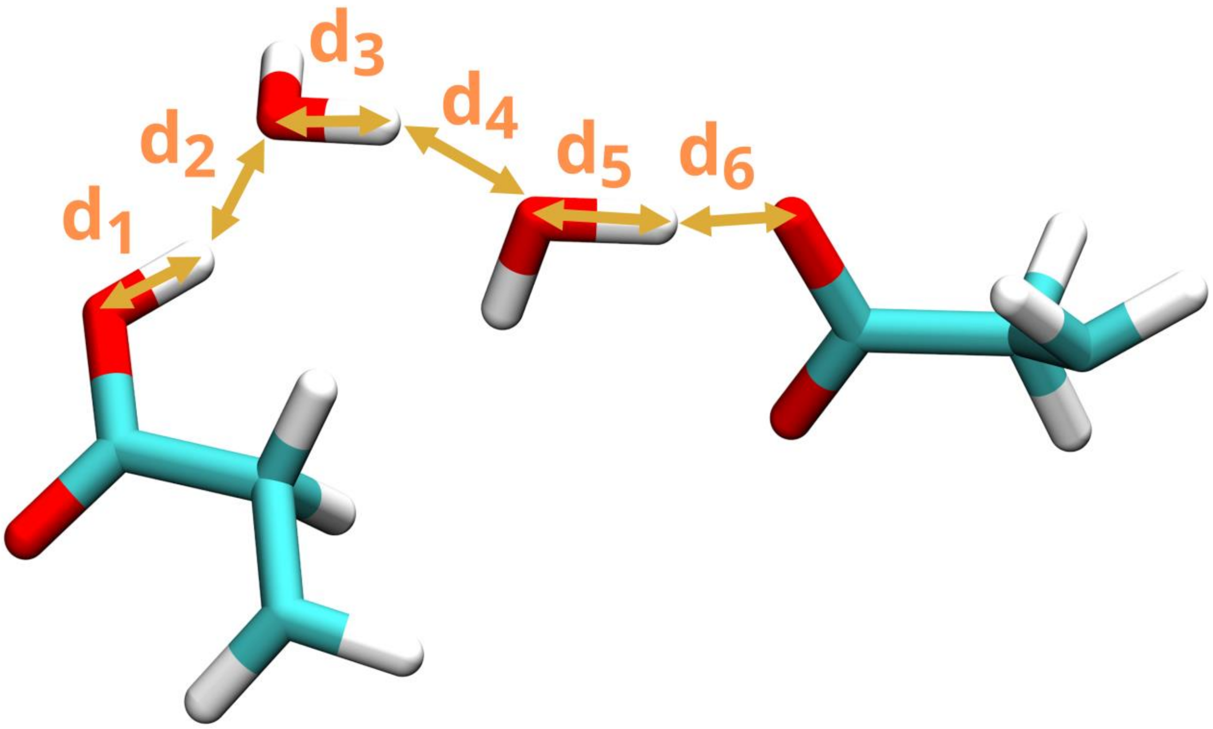

$$d = (d_1 - d_2) + (d_3 - d_4) + (d_5 - d_6)$$

Figure S11. Reaction coordinate applied in QM/MM free energy simulations.

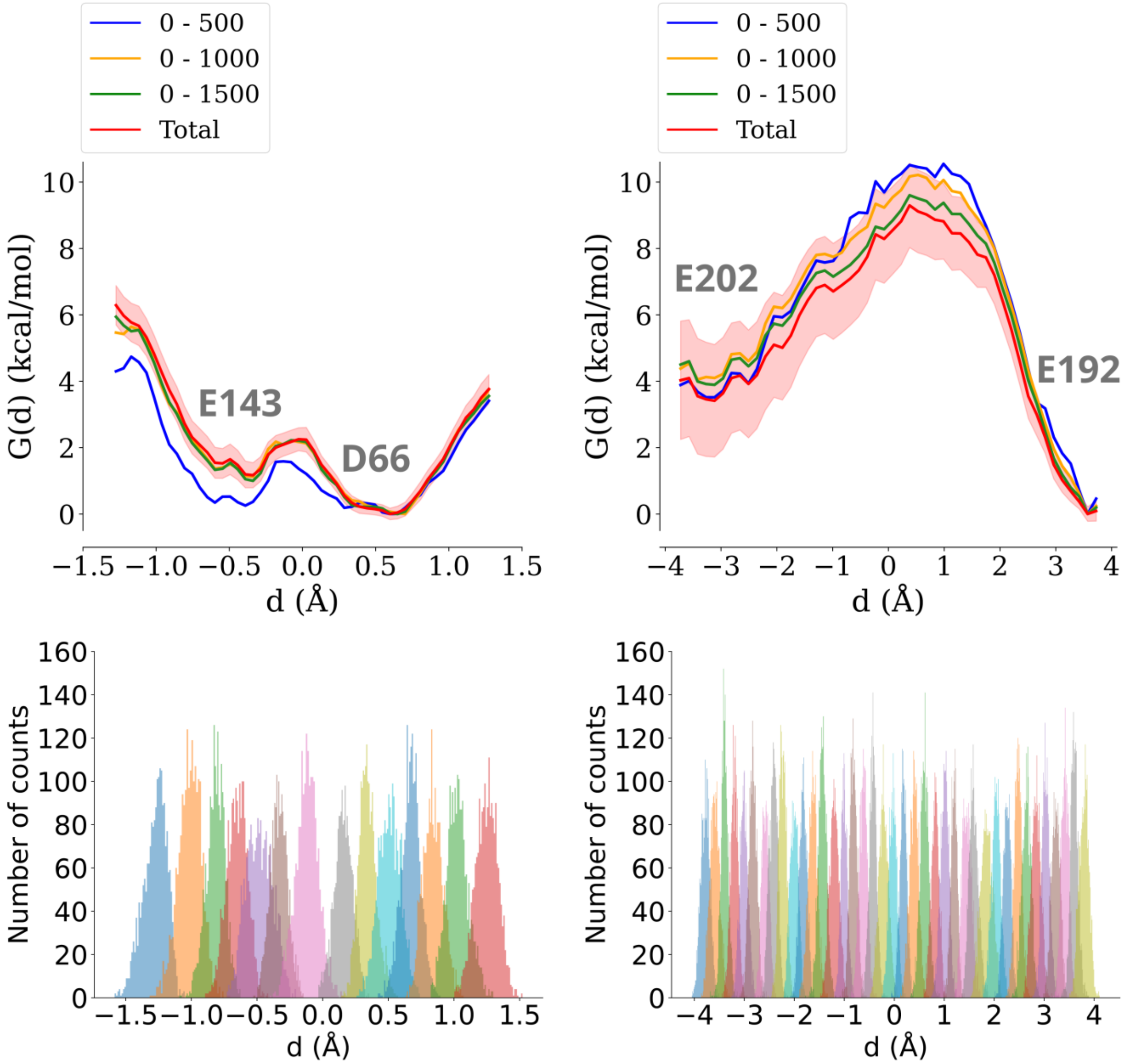

Figure S12. Convergence of free energy (G(d)) profiles and overlap of umbrella sampling histograms. Proton transfer between ^{ND1}Glu143 and ^{ND3}Asp66 (left; see also Table S1, setup L8) and from ^{ND1}Glu202 to ^{ND1}Glu192 (right; see Table S3, setup M5'). Top panels depict the free energy profiles, where lines of different colours mark the partial contributions from the first 500 fs (blue), 1000 fs (yellow), 1500 fs (green), and the full 2000 fs dataset (red). Shaded areas mark the bootstrapping errors of the overall free energy curve (see methods). The lower panels show the occupational histograms for the values of the reaction coordinate in the respective simulation windows.

Table S2. Simulated QM/MM setups L region of the E channel (see Fig. S1 B)

Setup	E70	E34	D66	E143	QM charge	E202	D199	E204	Unbiased trajectory (ps)	Umbrella sampling
L0	0	0	0	0	0	-1	-1	-1	5	-
L1	0	-1	0	0	-1	-1	-1	-1	2	-
L2	0	-1	0	0	-1	-1	0	-1	3	D66-E34
L3	-1	-1	0	0	-2	-1	-1	-1	2	-
L4	-1	-1	0	0	-2	-1	0	-1	5	D66-E34
L5	-1	-1	0	0	-2	0	-1	-1	5	D66-E34
L6	-1	-1	0	0	-2	-1	0	0	4	D66-E34
L7	-1	-1	0	0	-2	0	0	0	5	D66-E34
L8	0	0	-1	0	-1	-1	0	-1	5	E143-D66
L9	0	0	-1	0	-1	0	0	0	5	E143-D66
L10	-1	0	-1	0	-2	-1	0	-1	4	-
L11	0	0	-1	0	-1	-1	-1	-1	3	-
L12	0	-1	0	0	-1	-1	-1	-1	5	D66-E34
L13	0	-1	0	0	-1	-1	-1	-1	5	D66-E34

Table S3. Simulated systems for the M region of the E channel (see Fig. S1 C)

Setup	E143	Y142	E192	E227	QM charge	D66	E68	E202	D199	E204	Unbiased trajectory (ps)	Umbrella sampling
M0	0	0	0	0	0	0	0	-1	-1	-1	5	-
M1	-1	0	0	0	-1	0	0	-1	-1	-1	5	Y142-E143
M2	-1	0	0	0	-1	-1	0	-1	-1	-1	5	-
M3	-1	0	0	0	-1	0	-1	-1	-1	-1	5	-
M4	-1	0	0	0	-1	-1	-1	-1	-1	-1	5	Y142-E143
M5	-1	0	0	0	-1	0	0	-1	-1	0	5	Y142-E143
M6	-1	0	0	0	-1	0	0	0	-1	-1	5	Y142-E143
M7	-1	0	0	0	-1	0	0	0	0	-1	4	Y142-E143
M8	-1	0	0	0	-1	0	0	0	-1	0	5	Y142-E143
M9	-1	0	0	0	-1	0	0	0	0	0	5	Y142-E143
M10	-1	0	0	0	-1	0	0	0	-1	0	5	E192-E143
M11	-1	0	0	0	-1	0	0	0	0	0	5	E192-E143

Table S4. Simulated systems for the M' region of the E channel (constructed from a "flipped ^{ND1}Tyr142 conformation", see Fig. S1 D)

Setup	E143	Y142	E192	E227	E202	QM charge	D66	E68	D199	E204	Unbiased trajectory (ps)	Umbrella sampling
M1'	-1	0	0	0	0	-1	0	0	-1	0	5	E192-E143<br>E202-E192
M2'	-1	0	0	0	0	-1	0	0	0	0	5	E192-E143
M3'	-1	0	0	0	-1	-2	0	0	0	-1	5	E192-E143
M4'	0	0	-1	0	0	-1	0	0	0	-1	5	-
M5'	0	0	-1	0	0	-1	0	0	-1	-1	3	E202-E192<br>E143-E192

Table S5. Simulated systems for the R' region of the E channel (constructed from a "flipped ^{ND1}Tyr142 conformation", see Fig. S1 E)

Setup	E227	E202	D199	E204	R87	R274	QM charge	E143	E192	E206	Unbiased trajectory (ps)	Umbrella sampling
R1'	0	-1	0	-1	+1	+1	0	0	-1	-1	4.805	D199-E202
R2'	0	-1	0	0	+1	+1	+1	0	-1	-1	3.2	D199-E202

Table S6. Simulated systems for the R region of the E channel (see Fig. S9)

Setup	E192	E227	E202	D199	E204	R87	R274	QM charge	E143	E206	Unbiased trajectory (ps)	Umbrella sampling
R1	0	-1	0	-1	-1	+1	+1	-1	0	-1	4.6	-

References

[1] M. H. Olsson, C. R. Søndergaard, M. Rostkowski, and J. H. Jensen, PROPKA3: consistent treatment of internal and surface residues in empirical p K a predictions, Journal of Chemical Theory and Computation **7**, 525 (2011).

[2] D. N. Grba, I. Chung, H. R. Bridges, A.-N. A. Agip, and J. Hirst, Investigation of hydrated channels and proton pathways in a high-resolution cryo-EM structure of mammalian complex I, Science Advances **9**, eadi1359 (2023).
